# Early and diverse lipid consumption by *Saccharomyces cerevisiae*: an extensive targeted lipidomics approach and new perspectives for managing wine fermentation

**DOI:** 10.1038/s41538-026-00774-y

**Published:** 2026-02-28

**Authors:** Louise Ramousse, Jean-Paul Pais de Barros, Chloé Roullier-Gall, Hervé Alexandre

**Affiliations:** 1 Université Bourgogne Europe, Institut Agro, INRAE, UMR PAM, Dijon, France; 2 UMR1231, Inserm/Université Bourgogne Europe, Dijon, France; 3 Plateforme DiviOmics, US 58 BioSanD, Université Bourgogne Europe, Dijon, France

**Keywords:** Biochemistry, Biotechnology, Microbiology

## Abstract

Lipids play pivotal roles in yeast stress resistance, fermentation performance, and aroma formation. While fatty acid and sterol metabolism are documented, the fate of the broader lipidome during wine fermentation has been less investigated. We used targeted lipidomics to investigate lipid consumption kinetics in Chardonnay and Gewürztraminer musts fermented by three Saccharomyces cerevisiae strains (CX9, Fermol, and Finesse). We demonstrate that yeasts consume a broad range of exogenous lipids, including complex lipids like diglycerides and sterol esters, earlier than previously reported. The levels of 66 lipid species changed significantly during fermentation, with 21 species disappearing completely. This decrease was primarily driven by metabolic consumption; however, we also provided direct experimental evidence distinguishing passive adsorption from active uptake. We quantified the functional requirements for lipid uptake in oenological conditions: Free Fatty Acid (FFA) needs were matrix-dependent, whereas the phytosterol average specific consumption rate (6.73 ± 2.48 mg/L/10⁸ cells) was relatively constant across strains and musts. Finally, we observed that lipid consumption profiles were strongly correlated with their initial abundance in the must. Our results demonstrate that yeast lipid demand involves a wider diversity of molecules than previously thought, providing precise metrics to optimize nutrient supplementation strategies and improve yeast strain selection.

## Introduction

Lipids are essential compounds in the metabolism of *Saccharomyces cerevisiae*. In the context of oenology, the term “lipids” encompasses a vast and chemically diverse group of hydrophobic or amphipathic molecules. This category includes fundamental structural components such as sterols and fatty acids (both free and esterified), as well as complex lipids like glycerophospholipids (including mono-, di- and triglycerides), phospholipids, and sphingolipids (such as ceramides). They are stored in organelles or biological membranes and serve as a source of energy, structural elements, and signalling molecules^1,2^. They also play an important role in resistance to the stresses (temperature, anaerobiosis, nutritional and ethanol) induced by alcoholic fermentation, thereby contributing to the success of this process^3^. Beyond sugars, lipids, nitrogen, and vitamins are key components of yeast nutrition. Nitrogen nutrition is well characterized in oenology, and interest in vitamin nutrition is growing. Several studies have highlighted the essential role of unsaturated fatty acids and sterols in the growth and viability of *Saccharomyces cerevisiae* and in the production of aromas^4–10^. However, these lipid classes represent only a small fraction of the lipid diversity in grape musts^11^.

During alcoholic fermentation, sterols – ergosterol and its intermediates – and unsaturated fatty acids become essential nutrients for *Saccharomyces cerevisiae*, as their biosynthesis requires molecular oxygen^12^. During fermentation, oxygen becomes limiting, blocking the biosynthesis of these lipids. The yeast must then assimilate lipids from its external environment to ensure its survival. This assimilation of exogenous lipids by yeast is mediated by transporters such as the ABC transporters Pdr11 and Aus1, which are responsible for the uptake and transport of sterols. Fatty-acid transport remains a matter of debate. Some studies have reported the simple diffusion of fatty others^13,14^, whereas others have reported fatty acid transport mediated by a transport protein called Fat1p, followed by activation by FACS (fatty acyl-CoA synthetase)^15,16^. However, grape musts contain a wide variety of lipids, including di- and triglycerides, phospholipids and ceramides^11^. While the role of sterols and unsaturated fatty acids as anaerobic growth factors is well established, the relevance of other grape lipids—such as triglycerides, phospholipids, and ceramides—for yeast nutrition remains largely unexplored. Unlike fatty acids and sterols, *S. cerevisiae* is generally considered to lack the extracellular hydrolytic enzymes required to assimilate these complex lipids under oenological conditions. Consequently, few studies have investigated their fate during fermentation.

Nevertheless, given the transporters and lipid integration mechanisms described above, it is plausible that other classes of lipids in addition to sterols and fatty acids are consumed by *Saccharomyces cerevisiae*, an aspect that received limited attention in real oenological matrices.

Most of what we know relates to the influence of fatty acids and sterols on fermentation kinetics and the intracellular composition of yeasts^7,17^. There are currently few detailed time-series studies for the consumption of total lipids and other classes of lipids in musts. Studies on the intracellular lipid assimilation to date have been conducted in musts or synthetic media. Duan and coworkers reported that unsaturated fatty acid levels peak in the mid-exponential phase, providing an estimate as to the time point at which needs are most intense^18^. Conversely, maximum sterol assimilation has not been explicitly demonstrated. Nevertheless, traces of ergosterols act as a “spark” at the beginning of fermentation, initiating cell growth and, in the absence of oxygen, exogenous sterols are used during the growth phase^19^. Fatty-acid consumption has been reported to be matrix-dependent, whereas ergosterol assimilation appears to be strain-dependent^17,20,21^.

Kinetic studies of lipid uptake from grape must by yeasts are of both scientific and practical interest in oenology and biotechnology. Yeasts (especially *Saccharomyces cerevisiae*) use the lipids present in the must to build their cell membranes^3,22,23^. Lipid composition (fatty acids, sterols, etc.) influences membrane fluidity and permeability, thereby affecting the ability of the yeast to absorb sugars and tolerate alcohol^24–27^. Monitoring lipid uptake kinetics, therefore, provides insight into when and how yeasts mobilize these lipids. Lipid availability can determine the speed of fermentation and prevent stuck fermentations or slowdowns^17,28,29^. Kinetic studies can provide information about the critical moments at which a lipid deficit may slow fermentation, making it possible to optimize the potential supply of nutrients (oxygen, unsaturated fatty acids, sterols). Furthermore, lipids influence the biosynthesis of aromatic compounds (higher alcohols, esters, etc.). An understanding of their consumption can therefore help to establish correlations between lipid nutrition and the sensory profile of the wine. This essential information can help optimize yeast lipid nutrition, improving fermentation efficiency and enhancing wine stability and quality.

We therefore performed an extensive targeted lipidomics analysis to investigate lipid consumption by different yeasts in different musts. Unlike traditional methods, often limited to total fatty acid or sterol profiling, this high-resolution approach enables the simultaneous monitoring of specific lipid molecular species, including complex lipids. This level of detail is essential to accurately distinguish between active metabolic requirements and passive phenomena across the different lipid classes.

## Results

### Initial composition of the musts

Two white musts from Chardonnay and Gewürztraminer grapes were used for this analysis of lipid consumption by yeasts under oenological conditions.

IRTF analyses showed sugar contents of 190.8 ± 0.5 g/L for the Chardonnay and 200.5 ± 0.4 g/L for the Gewürztraminer must. Yeast assimilable nitrogen (YAN) were 280.4 ± 2.5 mg/L for the Chardonnay and 518.9 ± 3.4 mg/L for the Gewürztraminer must (Table 1). Regarding vitamins, concentrations of vitamin B3 were 924.9 ± 1.3 µg/L for the Chardonnay and 739.8 ± 1.8 µg/L for the Gewürztraminer must (Table 1). Lipid analysis revealed sterol esters concentrations of 281.7 ± 12.0 mg/L for Chardonnay and 676.0 ± 24.5 mg/L for Gewürztraminer. Sterols were quantified at 3.3 ± 0.2 mg/L for Chardonnay must and 4.1 ± 0.1 for Gewürztraminer must. Total fatty acids were measured at 18.9 ± 0.9 mg/L for Chardonnay and 23.3 ± 0.8 mg/L for Gewürztraminer. Other lipids, such as FFA, PC, PE, PI, CER, TGDG, and sterols were also quantified (Table 1). Finally, the sterol content of the musts used here is not consistent with a deficiency, ranging from 2 to 8 mg/L^17,28^ (Table 1).

**Table 1:** Classical oenological parameters, nitrogen content, vitamin concentrations and lipid composition of Chardonnay and Gewürztraminer grape musts prior to fermentation

		Chardonnay	Gewürztraminer
Classical Oenological Parameters			
pH		3.2 ± 0	3.5 ± 0
AT	(g/L)	4.3 ± 0	3.6 ± 0
D	(g/mL)	1.1 ± 0	1.1 ± 0
Sucre	(g/L)	190.8 ± 0.5	200.5 ± 0.4
TAP	(%)	11.3 ± 0	11.9 ± 0
AV	(g/L)	0.2 ± 0	0.1 ± 0
AMal	(g/L)	4.7 ± 0	4.2 ± 0
Tartaric	(g/L)	3.9 ± 0	3 ± 0
AGluc	(g/L)	2 ± 0	0.8 ± 0
Nitrogen			
Ammonium	(mg/L)	74.2 ± 2.2	180.3 ± 2.6
Primary Amino Nitrogen	(mg/L)	206.2 ± 1.6	338.6 ± 2.1
YAN	(mg/L)	280.4 ± 2.5	518.9 ± 3.4
Vitamins			
B2	(µg/L)	11.4 ± 0	40.4 ± 4.7
B3	(µg/L)	924.9 ± 1.3	739.8 ± 1.8
B6	(µg/L)	898.5 ± 14.8	105.3 ± 26.8
Lipids			
TFA	(mg/L)	18.9 ± 0.9	23.3 ± 0.8
FFA	(mg/L)	8.2 ± 0.2	10.6 ± 0.6
Sterols	(mg/L)	3.3 ± 0.2	4.1 ± 0.1
SE	(mg/L)	281.7 ± 12.0	676.0 ± 24.5
Cer	(mg/L)	4.5 ± 0.2	7.6 ± 1.3
TGDG	(mg/L)	61.7 ± 0.36	51.6 ± 9.9
PC	(mg/L)	79.8 ± 16.9	27.1 ± 2.8
PE	(mg/L)	43.0 ± 4.7	35.5 ± 2.7
PI	(mg/L)	80.9 ± 2.3	89.4 ± 10.9

*AT* titratable acidity, *D* density, *TAP* potential alcohol. *AV* volatile acidity, *AMal* malic acid, *N_Ass* assimilable nitrogen, *N_Amin* amino nitrogen, *N_NH₃* ammoniacal nitrogen, *AGluc* Gluconic acid, *TFA* Total Fatty Acids. *FFA* free fatty acids, *SE* sterol esters, *Cer* ceramides, *TGDG* tri-, di-acylglycerides, *PC* phosphatidylcholine, *PE* phosphatidylethanolamine, *PI* phosphatidylinositol, *YAN* yeast assimilable nitrogen. Qualitative changes in lipid levels during alcoholic fermentation

Values are expressed as mean ± standard deviation (*n* = 3).

We used three commercial strains of *Saccharomyces cerevisiae* to investigate the kinetics of lipid consumption by yeast. Each strain x must combination was fermented in triplicate. Fermentation, growth kinetics and wine oenological parameters are shown, together with the corresponding calculated parameters, in supplementary data figure S1 and tables S10 and S11. Five samples were taken during each fermentation to analyze the lipid content of the fermenting musts over time. We monitored a total of 94 lipids across 10 chemical classes. From this broad panel, we focused our kinetic analysis on four classes (FFA, sterols, SE, and DG), as these were the classes displaying the highest number of significantly impacted lipids during alcoholic fermentation. The four lipid classes selected for this preliminary study were those displaying the greatest changes in concentration during alcoholic fermentation

We investigated the impact of fermentation on the four lipid classes studied by performing a Student’s *t*-test (*p*-values < 0.05 considered significant) for each set of conditions (strain x must) and each lipid, to determine whether there was a significant difference in concentration between the start and end of the experiment. We found a significant difference for 66 of the 94 lipids in at least one strain x must combination and in two of three replicates (*p*-value < 0.05) (supplementary data: Table S12).

**Fig. 1:**
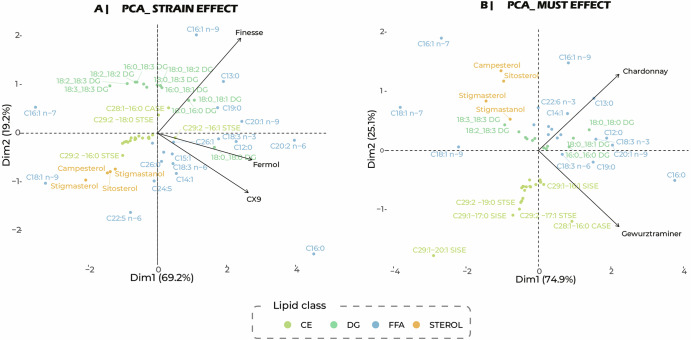
Analysis of the changes in the level of each lipid between the start and end of fermentation, focusing on the strain effect. **A** Principal component analysis by change in concentration (log_2_foldchange) from the start to the end of the fermentation, according to strain effect. Each point corresponds to a lipid, coloured according to its biochemical class. Volcano plots represent changes in lipid concentration between the start and end of the fermentation for each strain in the Chardonnay must. **B** Principal component analysis by change in concentration (log_2_foldchange) from the start to the end of the fermentation, according to must effect. Each point corresponds to a lipid, coloured according to its biochemical class. Volcano plots represent changes in lipid concentration between the start and end of the fermentation for each strain in the Chardonnay must. SE sterol esters, DG diglycerides, FFA free fatty acids, CASE campesterol esters, SISE sitosterol esters, STSE stigmastanol esters.

Principal component analyses (PCA) were performed to evaluate the effects of yeast strain (Fig. 1A) on the change in lipid concentration during fermentation. For the strain effect, the first principal component (PC1) explained a majority of the variance (69.2%). The loadings on this component primarily opposed saturated fatty acids to polyunsaturated DG and phytosterols. Along PC1, the three strains behaved similarly, revealing a shared pattern of lipid consumption pattern during alcoholic fermentation. By contrast, PC2 (19.2%) highlighted strain-specific differences. On the score plot, CX9 and Fermol clustered negatively, whereas Finesse displayed positive scores. The corresponding loadings indicated that this separation was driven by specific changes in DG levels. This finding indicates a common lipid response to fermentation (PC1) overlaid with a subtle strain-dependent signature (PC2). Although a difference was found between strains, confirmation with a larger number of more representative strains is required.

Another PCA was performed to evaluate the effects of the must matrix (Fig. 1B) on the change in lipid concentration during fermentation. PC1 accounted for 74.9% of the variance, capturing the lipid response common to both Chardonnay and Gewürztraminer, again mainly linked to saturated fatty acids. PC2 (25.1%) clearly separated the two matrices: Chardonnay was associated with loadings corresponding to phytosterols (campesterol, stigmasterol, sitosterol) and specific unsaturated fatty acids (C16:1 n-7, C18:1 n-7, C18:1 n-9), whereas Gewürztraminer was distinguished by changes in sterol ester concentrations (Supplementary Fig. S2D). Together, these PCAs reveal a conserved basis for lipid consumption patterns (PC1), modulated by both strain- and must-specific factors (PC2). As observed for strains, these observations for musts require confirmation with a larger panel of musts.

**Fig. 2:**
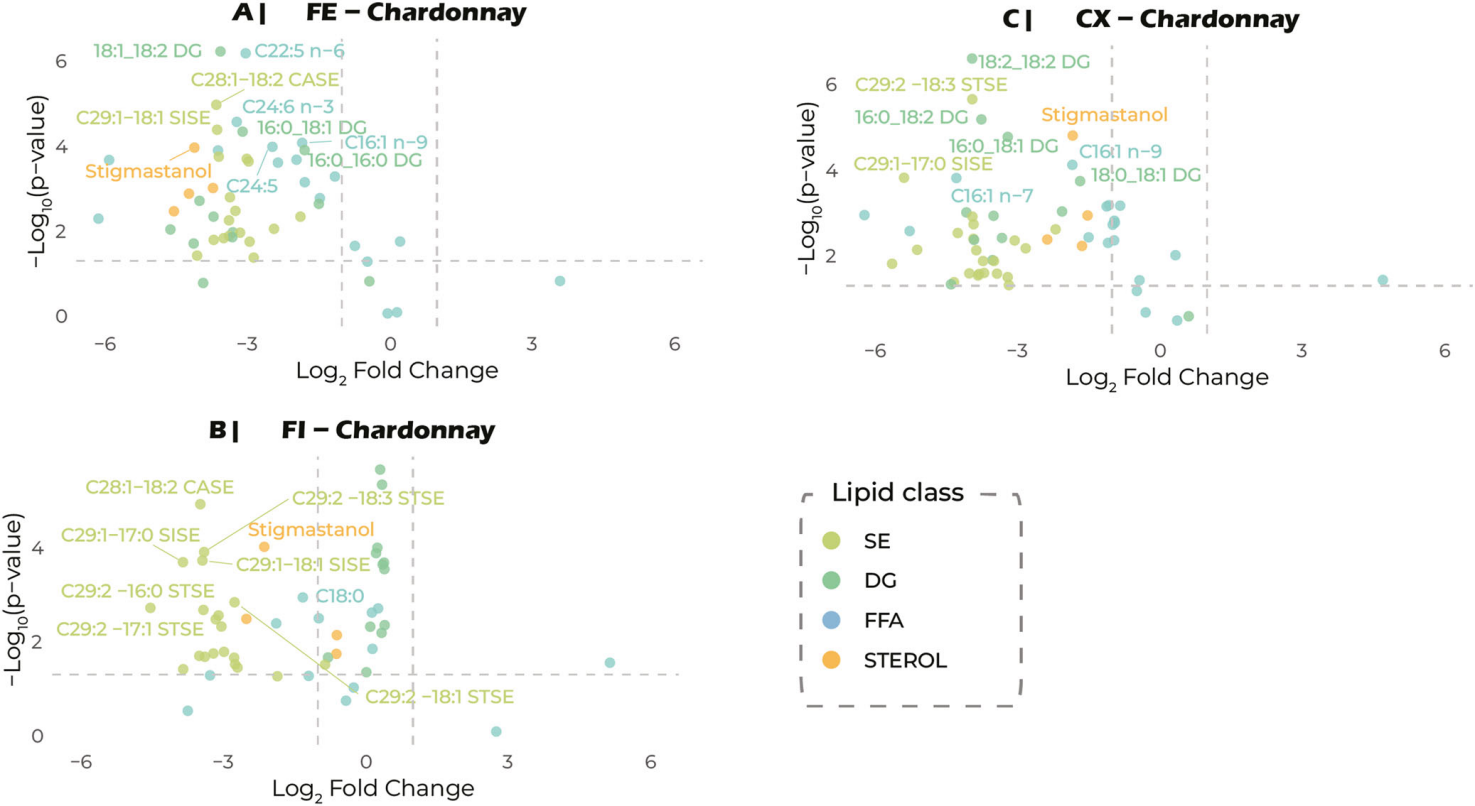
Analysis of the variations in the level of each lipid between the start and end of fermentation. Volcano plots represent changes in lipid concentration between the start and end of the fermentation for each strain in the Chardonnay must. **A** Fermol strain and Chardonnay must, **B** CX9 strain and Chardonnay must, and **C** Finesse strain and Chardonnay must. The x-axis indicates log_2_ fold change, and the y-axis statistical significance (-log_10_ *p*-value). Dotted lines delimit the thresholds of significance (*p* < 0.05) and biological change (|log_2_FC | > 1). The annotated lipids are those displaying the greatest change in concentration. SE sterol esters, DG diglycerides, FFA free fatty acids, CASE campesterol esters, SISE sitosterol esters, STSE stigmastanol esters.

Volcano plots confirmed a structured evolution of the lipid profile (Fig. 2A–C; Supplementary Fig. S2A–C). The number of significantly affected lipids remained remarkably stable and was little affected by the grape matrix or yeast strain. Two-thirds (44 lipids) of the 66 lipids affected in at least one combination displayed consistent changes in concentration in all conditions. Strain- and must-specific responses were observed for 18 lipids. Four lipids displayed exclusively (16:0_16:0 DG; C18:3n-6; C29:1-14:0 SISE; C29:2-17:0 STSTE) matrix-dependent responses. Interestingly, most of the lipids significantly affected by fermentation displayed a decrease in concentration. Only a limited number displayed an increase in concentration during fermentation; these lipids included the 11 DGs detected exclusively in Chardonnay with the Finesse strain, and 6 FFAs predominantly found in Chardonnay — except for C16:0, which was more abundant in Chardonnay with CX9, and in Gewürztraminer with CX9 and Finesse (Fig. 1).

### Quantification of lipid adsorption by inactivated yeast

We investigated the ability of yeasts to adsorb the lipids found to decrease in concentration during fermentation by incubating inactivated cells for 72 h in a must containing natural lipids, which we analyzed before and after exposure to the yeast.

**Fig. 3:**
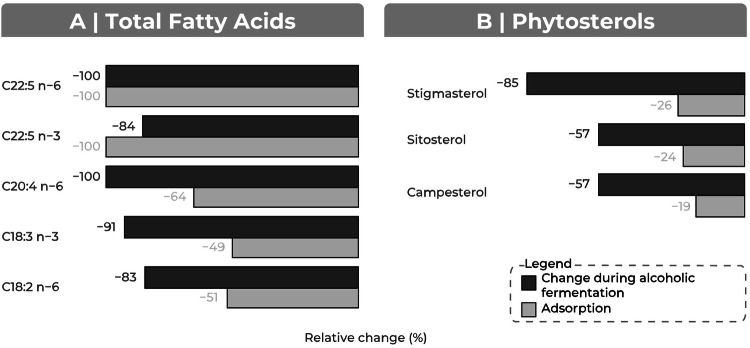
Comparative representation of the effects of adsorption and alcoholic fermentation on the concentrations of different lipids. The lipids shown are those displaying a significant decrease in concentration due to adsorption (*t*-test, *p*-value < 0.05), corresponding to 5 of 44 TFAs, 0 of 44 FFAs and 3 of 4 phytosterols. Each bar corresponds to the relative change (%) in concentration for a given lipid, according to two mechanisms: adsorption on yeast and after alcoholic fermentation. Negative values indicate a decrease in concentration during both processes. For each lipid, the concentration values shown are the mean of triplicate measurements, and the percentage change was calculated from these averaged values. Numerical annotations indicate the relative percent change. Lipids are grouped by biochemical class: (**A**) TFA (total fatty acids) and (**B**) sterols.

After incubation with inactivated yeast, most lipids displayed no significant change in concentration (44/44 FFAs, 39/44 TFAs, and 1/4 sterols; *p* > 0.05, *t*-test), indicating that they were not adsorbed during contact. These findings support the conclusion that the decrease in lipid levels observed during alcoholic fermentation primarily reflects metabolic consumption. Notably, none of the FFAs studied (C12:0–C26:1) displayed a significant change in concentration after incubation, further excluding adsorption as a mechanism in this experiment (Fig. 3).

During the time of contact studied here, the observed changes in certain lipid concentrations were surprisingly significant (Fig. 3). All three phytosterols displayed a moderate decrease in concentration due to adsorption, with changes in concentration ranging from –26% (stigmasterol) to –19% (campesterol) (Figs. 3 and S3 of supplementary data). Nonetheless, phytosterol concentrations decreased to a much greater extent during the course of alcoholic fermentation with active yeasts, with changes of –85% (stigmasterol) to –57% (sitosterol and campesterol) (Fig. 3).

Regarding total fatty acids, the decreases in C18:2 n-6, C18:3 n-3, and C20:4 n-6 levels during fermentation (changes of –83%, –91%, and –100%, respectively) were much greater than the corresponding decreases due to adsorption (changes of –51%, –49%, and –64%, respectively). For those with C22:5 n-6 and C22:5 n-3 chains, decreases in concentration during fermentation (–100% and –84%, respectively) closely matched those observed in adsorption experiments (–100% for both) (Fig. 3).

### Lipid consumption kinetics

**Fig. 4:**
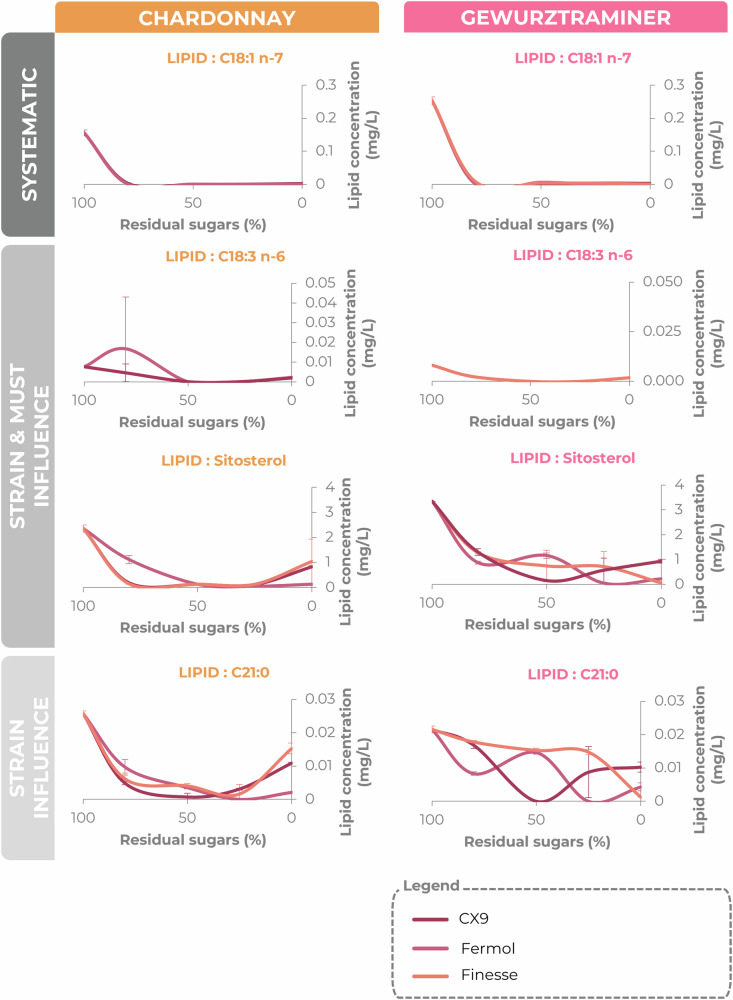
Changes in mean lipid concentration (mg/L) as a function of residual sugar (%) during the alcoholic fermentation of Chardonnay (left column) and Gewürztraminer (right column) musts. This figure includes only combinations in which lipid concentrations changed significantly during the course of fermentation (t-test, α=0.05). Each curve represents a strain: CX9 (burgundy), Fermol (pink) and Finesse (orange). The lipids chosen to illustrate the various influences encountered during the experiment are classified according to the type of influence observed. Systematic: similar profile regardless of strain or medium (e.g. C18:1 n-7); strain and matrix influence: variable profiles depending on grape variety and strain (e.g. C18 :3 n-6 and sitosterol); and strain influence: different profiles between strains regardless of grape variety (e.g. C21 :0). Error bars represent the standard deviation of the measured values (*n* = 3).

We followed lipid concentrations through the course of alcoholic fermentation. As adsorption tests confirmed that decreases in the levels of free fatty acids and phytosterols reflected metabolic consumption, we focused our kinetic analysis on these two lipid classes. The consumption of lipids displaying significant changes in concentration during fermentation (26 free fatty acids and 4 phytosterols; *t*-test, *p* < 0.05) was analyzed over three phases: exponential (100–80% residual sugars), stationary (80–25% residual sugars), and late fermentation (25–0% residual sugars)(Fig. 4 and S4–S10 of supplementary informations).

All four phytosterols studied, and 17 of the 44 free fatty acids — including 9 saturated, 5 monounsaturated, and 3 polyunsaturated free fatty acids — were completely consumed. Of particular interest, six of these free fatty acids — C15:0, C18:0, C20:0, C16:1 n-7, C18:1 n-7, and C20:1 n-7 — were fully consumed in every single scenario (Fig. 4).

### Exponential phase, preferred lipids

During fermentation, most of the lipids displaying significant changes in concentration were consumed quite early, especially during the exponential growth phase. In this phase, three free fatty acids — C15:0, C18:0, and C18:1 n-7 — were consistently depleted, regardless of the yeast strain or must used (see Fig. 4, and Supplementary Figs. S4 and S5). Other free fatty acids, including 11 FFAs (5 saturated, 4 monounsaturated, 2 polyunsaturated; for example, C26:0, C16:1 n-7, C18:3 n-6) were also consumed early, but in a strain- and must-dependent manner (see Fig. 4, Supplementary Fig. S5 and S9). Phytosterol consumption also varied with strain and must: total depletion was observed only in the Chardonnay must, with the CX9 and Finesse strains during the exponential phase, with potential adsorption rates of 19–26% (Fig. 3).

### Lipids consumed during the stationary phase

During the stationary phase, we can see the effects of different strains on lipid consumption. The Fermol strain completely depleted phytosterols in both musts right at the start of this phase. Similarly, strain CX9 consumed C13:0 and C21:0 in both musts, whereas Fermol completely exhausted the supply of C21:0 and C23:0. We also observed strain- and must-dependent effects on certain fatty acids, such as C16:1 n-7, C17:1, C18:3 n-6, C20:0, C20:1 n-7, C25:0, and C26:0. The lipids consumed towards the end of fermentation constitute only a small proportion of the total and they affected by the specific yeast strain and interactions with the must. For instance, strain CX9 used C17:1 in both types of must, whereas C16:1 n-7 was depleted only in Chardonnay must, but the strains CX9 and Fermol.

### Lipid requirements

We assessed the metabolic needs of the yeast in more detail by calculating specific consumption independently of adsorption rates and then normalizing by biomass. This method made it possible to quantify the lipid requirements of the yeast precisely under oenological conditions. For each combination and for each lipid displaying a significant change in concentration during fermentation, we determined the total specific consumption.

We report overall lipid consumption per strain and per matrix in Fig. 4. However, statistical analyses were conducted at the level of individual lipid species across the full dataset. This approach highlights intragroup effects and minimizes biases potentially arising from data aggregation.

### Fatty acids

**Fig. 5:**
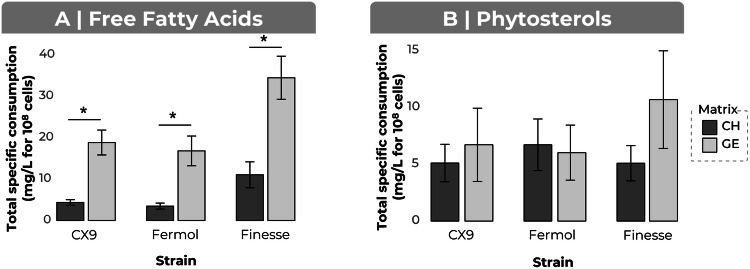
Effect of conditions on the specific consumption of free fatty acids and phytosterols. Fatty acids (**A**) and phytosterols (**B**). The strains were compared (CX9, Fermol, Finesse), as were the Chardonnay (CH) and Gewürztraminer (GE) matrices. The bars represent the sum of the total specific consumption averages calculated from the replicate means. * Differences between groups were assessed in a Kruskal-Wallis test (α = 0.05).

Total specific free fatty-acid consumption varied considerably between strains and matrices, from 34.5 ± 5.20 mg/L per 10⁸ viable cells for the Gewürztraminer-Finesse combination to 3.48 ± 0.735 mg/L per 10⁸ viable cells for the Chardonnay-Fermol combination. Statistical analyses (Kruskal–Wallis, with Dunn’s Bonferroni post-hoc correction, α = 0.05) revealed significant differences between musts, with higher consumption in Gewürztraminer (*p* = 7.716e−6). By contrast, no strain-dependent effect was detected. These findings indicate that free fatty-acid requirements depend on the must but not the yeast strain (Fig. 5A).

The most widely consumed free fatty acids were C16:1n-9, C18:1n-9, C22:6n-3, and C16:1n-7, with total consumptions of 13.78 ± 4.64, 3.70 ± 1.44, 1.36 ± 0.77, and 1.10 ± 0.75 mg/L per 10⁸ viable cells, respectively (see Table 2). Interestingly, these lipids were not always completely consumed during the fermentation process. C16:1n-9 was consistently available in all conditions, suggesting that it was present in surplus. By contrast, C18:1n-9 and C22:6n-3 levels differed between conditions, sometimes being limiting and sometimes abundant. However, C16:1n-7 was completely consumed in every scenario and was therefore a fully limiting substrate.

**Table 2:** Mean free fatty-acid consumption by yeast during alcoholic fermentation, expressed in mg/L per 10⁸ viable cells, by matrix (*p*-value = 7.76e−6). Values are presented as the mean ± standard deviation

Lipid	Mean total consumption ± standard deviation (mg/L per 10^8^ cells)	
	Chardonnay	Gewürztraminer
C12:0	0.04 ± 0	0.02 ± 0.01
C13:0	0.001 ± 0.001	0.01 ± 0
C14:1	0.03 ± 0.02	0.15 ± 0.02
C15:0	0.03 ± 0	0.03 ± 0.01
C15:1	0.04 ± 0.01	0.04 ± 0
C16:1 n-7	0.22 ± 0.04	1.55 ± 0.43
C16:1 n-9	10 ± 2.77	15.04 ± 4.54
C17:1	0 ± 0	0.62 ± 0.19
C18:0	0.21 ± 0.02	0.4 ± 0.11
C18:1 n-7	0.36 ± 0.02	0.74 ± 0.2
C18:1 n-9	2.61 ± 0.1	4.61 ± 1.39
C18:3 n-3	0 ± 0	0.47 ± 0.2
C18:3 n-6	0.01 ± 0	0 ± 0
C19:0	0 ± 0	0 ± 0
C20:0	0.02 ± 0	0 ± 0
C20:1 n-7	0.09 ± 0.02	0 ± 0
C20:5 n-3	0.22 ± 0.01	0.24 ± 0.02
C21:0	0.03 ± 0.01	0.05 ± 0.03
C22:6 n-3	0 ± 0	1.36 ± 0.77
C23:0	0.01 ± 0	0.02 ± 0.01
C24:6 n-3	0.38 ± 0.04	0.62 ± 0.35
C25:0	0.03 ± 0.03	0.04 ± 0.02
C26:0	0.39 ± 0.07	0.25 ± 0.06
Total	14.7 ± 2.25	26.3 ± 3.40

### Phytosterols

Total specific sterol consumption varied slightly between strains and matrices, from 10.7 ± 4.29 mg/L per 10⁸ viable cells for the Gewürztraminer-Finesse combination to 5.08 ± 1.55 mg/L per 10⁸ viable cells for the Chardonnay-Finesse combination, with additional variability due to interspecies differences in phytosterol use. However, statistical tests (Kruskal–Wallis, Dunn’s Bonferroni post-hoc test, α = 0.05) detected no significant effect of must (*p* = 0.4813) or strain (*p* = 0.4265). These results suggest that phytosterol requirements are largely independent of both strain and must (Fig. 5.B). Sitosterol was the most consumed phytosterol, at 5.38 ± 3.67 mg/L per 10⁸ viable cells (Table 3). Finally, sitosterol is the dominant phytosterol in grapes, accounting for 75–90% of total sterols in berries^20,30^. Exogenous β-sitosterol, campesterol, and stigmasterol are known to be incorporated by yeast in proportions reflecting their concentrations in the medium^7^, consistent with our observation that β-sitosterol was the most consumed phytosterol in our experiments.

**Table 3:** Mean phytosterol consumption by yeast during alcoholic fermentation, expressed in mg/L per 10^8^ viable cells

Lipid	Mean total consumption ± standard deviation (mg/L for 10^8^ cells)
Campesterol	0.170 ± 0.146
Sitosterol	5.38 ± 3.67
Stigmastanol	0.802 ± 0.320
Stigmasterol	0.378 ± 0.161
Total	6.73 ± 2.48

Values are presented as the mean ± standard deviation.

### The dose-response effect

The rates of lipid consumption by yeast were not affected by the matrix. However, the most consumed lipids were those with the highest initial concentrations. We investigated whether initial lipid availability drove specific consumption by constructing a linear model by lipid class (FFA and phytosterols) relating initial concentration to specific consumption.

**Fig. 6:**
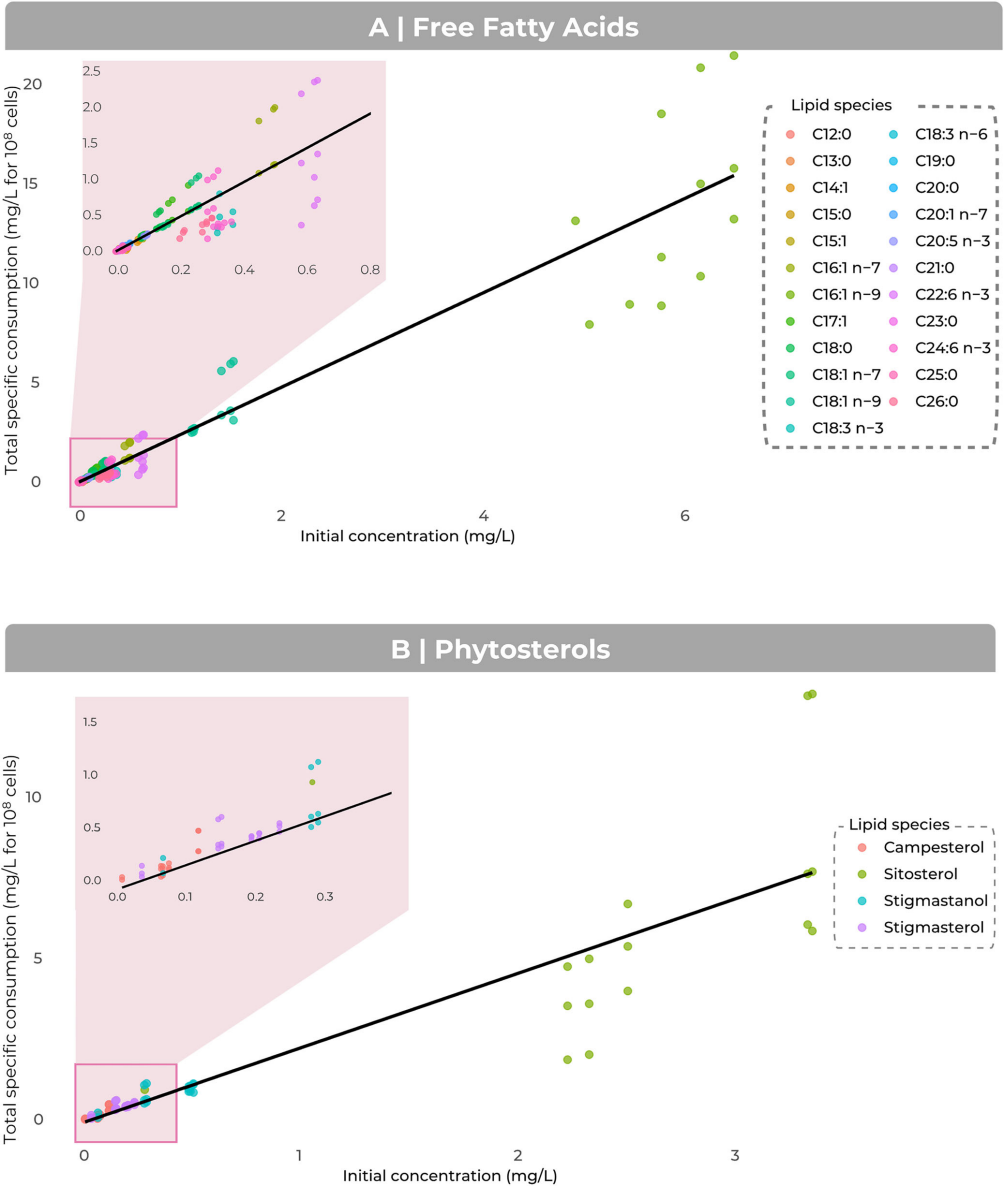
Relationship between initial lipid concentration and total specific consumption by cells. The panels show linear correlations between initial concentration (mg/L) and total specific consumption (mg/L for 10⁸ cells) for **(A**) free fatty acids and (**B**) phytosterols. The coloured dots represent lipid species. The trend curves (black lines) were adjusted by linear regression (the lm method). Magnifications are provided to improve visualization at low concentrations.

For free fatty acids (FFAs), the linear model revealed a very strong correlation between initial concentration and specific consumption by yeast. The coefficient of determination (R² = 0.91) indicates that 91% of the variability in consumption is explained by initial FFA levels. This highly significant relationship (*p* = 2e−16), with a slope of 2.37 ± 0.05 (Fig. 6).

Similarly, the linear model for phytosterols revealed a strong and significant correlation (R² = 0.81, *p* = 2.2e−16), explaining 81% of the variability in specific consumption, with a slope of 2.31 ± 0.14. The consumption of phytosterols by yeast is, therefore, also proportional to their initial concentration (Fig. 6).

## Discussion

The composition of the musts used in this study presented specific features relevant to fermentation kinetics and biomass production. While sugar levels indicated a standard potential for alcohol release, Yeast Assimilable Nitrogen (YAN) was well above the 140 mg/L deficiency threshold for both grape varieties. Conversely, vitamin concentrations were in the lower range of values reported in a recent study^31^. Specifically, vitamin B3 levels fell within the deficiency range (100–2000 µg/L) defined by Duncan and coworkers^32^, which could potentially limit fermentation rates or biomass yield. Regarding the lipid profile, sterol ester concentrations were notably higher than those we previously reported in white musts (0.12 to 56.51 mg/L)^11^. In contrast, TFA concentrations were lower than the ranges reported by Tumanov and coworkers (50 to 2880 mg/L)^11^. However, sterol content was consistent with standard requirements and did not present a deficiency risk (range 2–8 mg/L)^17,28^. Other lipid classes (FFA, PC, PE, PI, CER, TGDG) were present at concentrations within previously reported ranges.

Our findings reveal that fermentation triggers a robust and predictable biochemical response, characterized by a “core lipid signature” involving conserved metabolic pathways. The dual pattern of conserved and specific responses observed in the volcano plots opens up new avenues for the targeted modulation of lipid profiles. Specifically, the differential consumption of fatty acids by different strains could directly influence the intracellular pool of precursors available for the synthesis of ethyl esters, key contributors to the fruity aromatic profile of white wines^33^. Furthermore, the ability of specific strains to efficiently scavenge phytosterols from the must matrix (as seen with the Finesse strain) could be leveraged to enhance fermentation security in high-sugar musts, thereby preventing the sensory defects associated with stuck fermentations. Consequently, understanding these consumption kinetics offers a new criterion for selecting yeast strain x grape variety pairings to optimize both the sensory complexity and the technological robustness of the final wine.

Regarding the lipids that increased during fermentation, the absence of extracellular lipases in S. cerevisiae rules out active hydrolysis of triglycerides in the must by the yeast. Consequently, the atypical accumulation of diglycerides (DG) in the medium for the Finesse - Chardonnay modality suggests passive release. This phenomenon probably indicates early alteration of membrane integrity (ethanol stress or nutritional deficiency).

Alternatively, stress responses during the stationary phase may lead to lipid release promoted by membrane perturbations. As fermentation advances, increasing cell mortality^28,34^ and rising ethanol concentrations disrupt membrane lipid composition^27,35–37^ and enhance permeability^25^, ultimately facilitating the leakage of intracellular lipids into the medium^38^. Similarly, the increase in saturated fatty-acid levels with fermentation may reflect passive diffusion driven by concentration gradients^13,14^.

In this context, polyunsaturated fatty acids, which are not synthesized by yeast, serve as robust markers of exogenous consumption. The overall decrease in lipid concentrations during alcoholic fermentation is most plausibly explained by the consumption of the lipids concerned by the yeast. Previous studies have established that yeast actively metabolizes fatty acids and sterols^7,16^. Our results extend this observation by showing that diglycerides (DG) and Sterol Esters (SE) also decrease in concentration, a phenomenon not previously reported. Such decreases probably reflect metabolic use, but an alternative explanation involves adsorption onto the cell wall, as suggested in previous studies^39,40^. We therefore investigated whether adsorption could account for the observed lipid loss. To do so, we monitored adsorption using Sterols and Total Fatty Acids (TFA). Since TFA quantification encompasses the fatty acid moieties of all acylated lipids (including FFA, DG, and SE), this metric served as a robust global proxy to rule out adsorption for these decreasing classes.

*Saccharomyces cerevisiae* is widely recognized for its strong adsorption capacity towards hydrophobic molecules. Its biomass, whether inactive, living, or dead, can bind diverse molecules in solution^41^. This property has been exploited in winemaking to reduce the levels of undesirable compounds such as phenolics, sulfur derivatives, aromatics, and medium-chain fatty acids (C6–C11)^40–47^. Previous studies of fatty-acid adsorption have focused mainly on yeast hulls, with equilibrium reached within 24 h and unaffected by longer contact^39,40^. By contrast, equilibrium times vary depending on the substrate: 2–4 h for 4-ethylphenol^46^ and from minutes to hours for heavy metals^48–50^. We chose to use a 72 h contact period to ensure a complete and stable equilibrium. Given the differences in size and polarity between free fatty acids, total fatty acids (including complex lipids), and sterols, different adsorption mechanisms would be expected and prolonged contact increases the likelihood of capturing these interactions. While previous studies have highlighted the importance of the adsorption of short- and medium-chain fatty acids (C6–C12), such as hexanoic, octanoic, decanoic, and dodecanoic acids, due to their ethanol-dependent toxicity and the ability of yeast walls to bind them^39,40,51,52^. While the metabolic consumption of long-chain polyunsaturated fatty acids (such as linoleic acid) is well documented in oenology, the specific contribution of passive adsorption for FFAs with more than 12 carbons has been little investigated, probably due to the lower toxicity of these molecules and their assimilation by active yeast through either thermodynamic diffusion^13,14^ or protein-mediated uptake^15,16^. Consistent with this conclusion, Moonjai and coworkers demonstrated the uptake of linoleic acid (C18:2) by spheroplasts and its incorporation into lipid fractions, excluding cell-wall adsorption^53^. Together, these findings suggest that long-chain fatty acids are preferentially transported into the cell rather than binding to the yeast wall.

Regarding sterols, while the metabolic assimilation of phytosterols is well established, our findings provide direct experimental evidence quantifying the specific contribution of passive adsorption to the overall loss of phytosterols from the must. However, the difference implies that adsorption alone is insufficient to explain the decreases in concentration observed. Indeed, sterol depletion seems to be due essentially to active uptake by yeast, via the ABC transporters Aus1 and Pdr11^54^, consistent with the essential role of sterols in ethanol stress in addition to membrane integrity and fluidity.

As for total fatty acids, the lack of change for FFAs indicates that the reported values reflect changes in complex lipids. The decrease during fermentation results from a combination of metabolic and physicochemical mechanisms. While adsorption may contribute, the fact that decreases in C18:2, C18:3, and C20:4 are much greater than adsorption suggests that metabolic consumption is the main cause. Metabolically active yeast membranes may provide more interaction sites for complex lipids than inactivated cells^38,42,43^, or ethanol stress may facilitate lipid translocation. Chemical or enzymatic transformation is unlikely, as only a single potential precursor increased.

Thus, metabolic consumption is the main driver of the decrease in phytosterol and free fatty-acid concentrations. Consistent with previous reports, our data reconfirm that the decrease in lipid levels involves active metabolic pathways rather than simple physical adsorption^7,52^. However, our study adds specific quantitative evidence extending this conclusion to complex lipids (Diglycerides and Sterol Esters).

The complete depletion of specific saturated and monounsaturated FFAs (C15:0, C18:0, C20:0, C16:1 n-7, C18:1 n-7, and C20:1 n-7) suggests they play a vital role in yeast metabolism, acting as building blocks for membranes or sources of energy. The consistent use of these lipids across different strains and environments highlights a fundamental metabolic requirement under winemaking conditions.

In the exponential phase, the early depletion of saturated FFAs like C15:0 and C18:0 suggests they serve as substrates for β-oxidation, essential for synthesizing complex lipids for new membranes. In contrast, C18:1 n-7, which requires more enzymatic activity for oxidation, is likely incorporated directly into membranes^53^. Notably, the complete disappearance of these FFAs contrasts with earlier studies in wine matrices^55–58^ a difference likely due to sampling times, analytical techniques (LC-ToF MS, UPLC-MS/MS, GC-FID), or the use of red varieties like Carménère, which are richer in lipids^59^.

The timing of lipid consumption is also linked to environmental factors. The breakdown of unsaturated free fatty acids (FFAs) and phytosterols coincides with a drop in oxygen levels at the start of fermentation. Since acyl-CoA Δ9-desaturase and ergosterol production are oxygen-dependent^60–62^, yeast must obtain these compounds from the extracellular medium. The amount of sterols required for optimal growth ranges from 2 to 5 mg/L, depending on how much nitrogen is available^17^. Moreover, the addition of external phytosterols can greatly influence fermentation rates, the behaviour of yeast populations, and nitrogen absorption, highlighting the crucial role of these lipids in metabolism and overall health^5,30^. The uptake of sterols is facilitated by ABC transporters, such as AUS1 and PDR11, which are controlled by transcription factors, such as Upc2p^54^.

During the stationary phase, the observed strain-specific patterns —particularly for phytosterols—may reflect differing metabolic requirements or ethanol stress tolerance. As these lipids are essential for reinforcing stressed membranes, their uptake likely mirrors the specific physiological responses of each strain^20,27^. Conversely, long-chain saturated lipids can be converted into storage forms, such as sterol esters, fatty acid esters, or triglycerides, by acyltransferases, accumulating in lipid droplets as energy reserves^63^. Similarly, the disappearance of phytosterols was dependent on strain and must, with the Finesse strain completely depleting phytosterols in the Gewürztraminer must. These findings suggest that different yeast strains may have unique lipid requirements and survival strategies. The way lipids are consumed varies significantly with the type of lipid, the yeast strain, and the must, indicating that metabolic needs can differ. Future studies should focus on quantifying lipid requirements to improve our understanding of the needs of the yeast in winemaking conditions.

Our quantitative assessment provides a precise view of lipid requirements under real winemaking conditions. The high consumption of C16:1n-9 and C18:1n-9 is not particularly surprising given their essential roles. Palmitoleic acid and oleic acid are crucial components of *Saccharomyces cerevisiae* membranes involved in the maintenance of membrane integrity and fluidity, particularly in conditions of ethanol stress or in anaerobic conditions. Higher levels of oleic acid have also been linked to better ethanol tolerance^26,64^. *S. cerevisiae* cannot synthesize polyunsaturated fatty acids such as C22:6n-3 as it lacks the necessary ∆12 and ω3 desaturases^18^, but it can take these lipids up from the medium and use them as a sole carbon source for growth^65^.

Regarding phytosterols, we quantified the average sterol requirement at 6.73 ± 2.48 mg phytosterols/L per 10⁸ viable cells. Comparing this value to the initial sterol content of our grape must (3.3 to 4.1 mg/L, it appears that sterols may be a limiting factor in many standard fermentations. This observation provides direct scientific support for the common winemaking practice of sterol supplementation to prevent stuck fermentations.

Sterols are essential for yeast growth and viability during fermentation^66,67^. Previous studies estimated yeast sterol requirements at about 5 mg/L under standard nitrogen conditions^17^, with 2–8 mg/L phytosterol sufficient for normal growth, depending on the strain^28^, and 5 mg/L supporting biomass formation under anaerobiosis^7^. These studies used synthetic musts, which may not capture the complexity of natural grape must. By contrast, we used real grape musts in this study, providing a more accurate assessment of phytosterol consumption during alcoholic fermentation. Our results indicate a conserved sterol consumption profile across strains, although previous studies have reported strain-dependent effects on sterol and nitrogen assimilation^5,17^. By contrast to these previous studies, which focused on ergosterol or β-sitosterol, our analysis included a broader range of sterols, revealing potential compensatory adjustments between species resulting in a relatively stable per-cell consumption. From a practical standpoint, since sterol uptake occurs mainly in the exponential phase, winemakers should prioritize early nutrient supplementation to effectively support cell growth. These quantitative results highlight the lipids most strongly mobilized by yeast. While the consumption profile generally mirrored the initial abundance (with β-sitosterol being the most consumed), this behaviour is intrinsically linked to the overall must matrix and specifically the nitrogen/lipid balance. The high nitrogen content in our musts likely drove high biomass production, thereby mechanically increasing the absolute demand for the most abundant lipid resources available to sustain membrane synthesis.

A key finding of our study is the strong correlation between initial availability and assimilation, which suggests an almost proportional relationship between availability and assimilation. No maximum consumption threshold was observed under these conditions, indicating a dose-response effect consistent with passive or unregulated uptake. For free fatty acids, this pattern is consistent with passive diffusion models, in which non-esterified FFAs integrate into the plasma membrane according to their hydrophobicity and are then redistributed among membrane phospholipids^13,14,65^. Transport proteins, such as Fat1p and FACS, may be involved, but passive diffusion appears to dominate under the conditions tested^16^. Unlike FFAs, this dose-response pattern was unexpected for sterols, which are typically transported via regulated ABC transporters, notably *SUT1*^66,68^. The observed proportionality suggests that, under our experimental conditions, phytosterol uptake is limited by availability in the medium rather than by transport saturation.

It is also important to consider the nutritional context of these fermentations. Lipid consumption is closely linked to the nitrogen content of the must, as nitrogen availability influences biomass production and growth rate. In our study, both grape musts had high concentrations of yeast-assimilable nitrogen (YAN): 280 mg/L for Chardonnay and 518 mg/L for Gewürztraminer. These high nitrogen levels likely promoted vigorous growth and high peak population densities, thereby maximizing the demand for lipid incorporation into new cell membranes. Consequently, the high specific consumption rates observed here could partly reflect this nitrogen-rich environment. The differences in lipid consumption between the two musts are likely driven by their nitrogen content. The Gewürztraminer must, characterized by an exceptionally high YAN (518 mg/L), supported a higher maximum population (Nmax) compared to the Chardonnay. Since sterols and fatty acids are essential building blocks for plasma membranes, the higher biomass production in Gewürztraminer resulted in a higher total uptake of exogenous lipids. Additionally, the significantly higher consumption of free fatty acids observed in Gewürztraminer could be attributed to its specific composition. The higher initial sugar content (200.5 g/L compared to 190.8 g/L for Chardonnay) imposes greater osmotic stress on the yeasts. This stress is known to trigger membrane remodelling and increased fatty acid turnover to maintain membrane fluidity and integrity, which could explain the higher absorption rates observed. The robust linear model results provide a strong foundation for the application of machine learning to fermentation management. Our dataset, encompassing 94 lipids, provides a quantification of functional requirements and kinetic profiles, and is suitable for multivariate predictive approaches. Tools, such as random forests or artificial neural networks^69^, could be used to predict key fermentation parameters — including kinetics, biomass production, and final wine lipid profiles — from initial lipid concentrations and strain. Similar applications have been reported for lipid production in *Yarrowia lipolytica* and fermentation parameter prediction with decision trees or the Artificial Bee Colony algorithm^70–72^.

Moreover, this quantification of FFA and phytosterol requirements could support optimized must supplementation, with the use of machine learning to predict its impact on wine quality and design personalized supplementation strategies. This illustrates how yeast lipid assimilation, involving both passive diffusion and regulated protein-mediated uptake, can be integrated into predictive models for the optimization of fermentation. Future studies should explore a broader range of strains and musts, and consider lipid interactions during co- or sequential inoculations, which are common in oenology.

In conclusion, we applied a comprehensive targeted lipidomics approach to monitor the kinetics of 94 lipid species during fermentation. Our results corroborate the central metabolic role of Free Fatty Acids (FFAs) and phytosterols, while extending this observation to complex lipids such as Diglycerides and Sterol Esters, which consistently decreased regardless of the yeast strain or must matrix. We clarified the mechanisms involved: while metabolic consumption is the primary driver of lipid depletion, we provided experimental evidence quantifying the specific contribution of passive adsorption for phytosterols. Lipid mobilization occurred primarily during the exponential growth phase. By analyzing these kinetics, we determined specific lipid requirements to sustain fermentation in oenological conditions: while FFA needs were matrix-dependent (ranging from 14.7 ± 2.25 to 26.3 ± 3.40 mg/L per 10⁸ cells), phytosterol consumption was remarkably constant across all conditions (6.73 ± 2.48 mg/L per 10⁸ cells). Furthermore, we observed a strong dose-response relationship (R^2^ > 0.8) for both FFAs and phytosterols, indicating that consumption is driven by the interplay between initial abundance and yeast biomass demand. These quantitative findings pave the way for the creation of predictive models for fermentation performance, facilitating the development of targeted strategies for precision lipid supplementation.

## Methods

### Yeast strains

We studied the fermentation of the musts described above with three commercial oenological strains of *Saccharomyces cerevisiae*: FERMOL® Chardonnay (AEB, Kaysersberg, France), Oenoferm® Finesse (Erbslöh, Servian, France) and ZYMAFLORE® CX9 (Laffort, Bordeaux, France). The active dry yeasts (ADY) were rehydrated for 30 minutes in mineral water at 37°C.

### Grape musts

#### Main oenological characteristics

Two white musts of German origin, made from Chardonnay and Gewürztraminer grapes, were used in this study of lipid consumption by yeast. These matrices were selected to investigate lipid consumption in a clarified medium, preventing the extraction of lipids from grape skins that typically occurs during red wine maceration. The classical oenological parameters (sugar concentration, total acidity, volatile acidity, gluconic acid content, tartaric acid content, malic acid content, pH, potential degree of alcohol, and density) of these musts were measured by Fourier transform infrared spectroscopy (FTIR) with a WineScan™ apparatus (FOSS Electric, Denmark). We used 10 mL of the supernatant obtained by centrifuging the musts for 5 min at 9000 x *g* for these measurements, which are presented in Table S10 of the supplementary data. Assimilable nitrogen (amino acids and ammonium) was also determined on 200 µL of supernatant with a Y15 automatic enzyme analyzer (Biosystems, Barcelona, Spain) and the ammonia (reference: 12809) and primary amino nitrogen (reference: 12807) kits.

#### Vitamin quantification

Finally, the concentrations of vitamins B2, B3, and B6 and the corresponding vitamers were determined by High -Performance Liquid Chromatography (HPLC) by the following procedure. Prior to analysis, samples were centrifuged at 6800 x *g* for 10 minutes. The analytical method was adapted from the one originally described by Evers and colleagues^31^. An Agilent 1260 Infinity II LC system (Agilent, Santa Clara, CA, United States of America) was used for the analysis. This system featured a quaternary pump (Reference Agilent G7111B), an autosampler (G7129A), a Diode Array Detector (DAD) (G7115A), and a Fluorescence Detector (FLD) (G77121B). Data collection and analysis were managed using OpenLab CDS software (Agilent, Santa Clara, CA, USA). Ten external calibration standards, containing all vitamers at concentrations expected in grape musts, were prepared. Separation was achieved using a Zorbax SB-Aq column (4.6 × 250 mm, 5 µm), safeguarded by a Zorbax SB-Aq guard column (4.6 × 12.5 mm, 5 µm) (Agilent, Santa Clara, CA, USA). The column temperature was maintained at 25°C. The injection volume was 10 µL, and the flow rate was kept constant at 0.6 mL/min through the run. The specific gradient programme is detailed in the Supplementary data Table S9). A dual-detection approach, utilizing both DAD and FLD in series, was employed. The UV Detection (DAD): Five specific wavelengths (210 nm, 250 nm, 260 nm, 270 nm, and 290 nm) were applied to monitor each vitamer, exploiting their respective absorption maxima. The fluorescence detection (FLD). The FLD was used in line with the UV detector. The natural fluorescence of the vitamin B6/pyridoxine vitamers (λexitation = 320 nm; λemission = 370 nm) and vitamin B2/riboflavin (λexitation = 270 nm; λemission = 525 nm) was monitored. This data, combined with retention time and adsorption spectra, was used to confirm peak identity.

After these initial characterizations, the musts were immediately frozen and stored at −20°C until the fermentation experiments. Freezing is a standard preservation method in oenological research, known to maintain the stability of primary metabolites (sugars, nitrogen) and lipid profiles, ensuring that the fermentation substrate remains representative of the characterized matrix.

### Fermentation monitoring

#### Cell growth/Cell viability

The musts were then inoculated with each rehydrated yeast separately, at a density of 1 × 106 viable cells/mL by flow cytometry using cFDA (5-6-carboxyfluorescein diacetate, concentration of 1500 µM in acetone) (Invitrogen, ThermoFisher Scientific, Illkrich, France) staining method. Fermentations were carried out in 250 mL Schott flasks (Merk, Darmstadt, Germany) containing 200 mL of inoculated must sealed with DURAN® Membrane Venting Screw Caps (DWK, Wertheim, Germany). Each assay was performed in biological triplicate at 20°C without shaking. The end of fermentation was defined as the time point at which 2 g/L sugar remained in the sample.

#### Samplings

The samples (1 mL) were collected every 6 hours before reaching the stationary phase, then every 24 hours until the end of fermentation. Cell viability and population density were assessed by flow cytometry using the CFDA staining method. 2 µL of this fluorescent probe was added to 200 µL of cell suspension diluted in Mac Ilvain buffer (100 mM citric acid, 200 mM Na2HPO4, pH 4) for measurement. The samples were placed in the dark for 10 min before the analysis. The concentration of viable cells was obtained with the flow cytometer ThermoFisher Attune NxT (ThermoFisher Scientific, Waltham, USA). The Forward Scatter Height (FSH) threshold was set at 4,000. Cell excitation (autofluorescence) was performed using a 488-nm wavelength blue laser. CFDA fluorescence was detected using the BL1-A band-pass filter (530/30 nm), and the data for side-scatter light (SSC) and fluorescence intensity were analyzed. The fermentation rate was monitored using the method described by Seguinot and colleagues^73^, that is, by weighing the Erlenmeyer flasks to determine the mass lost corresponding to CO2 production during fermentation. The maximum fermentation rate was then calculated by deriving CO2 production over time. The endpoint of the fermentation was determined using the automatic enzymatic analyzer Y15 (Biosystems, Barcelona, Spain) with D-Glucose/D-Fructose reagent (Biosystems, Barcelona, Spain). The samples were centrifuged for 5 minutes at 9000 *× g* before analyzing the classical parameters of the finished wines using FTIR and measuring the volatile compounds. During the alcoholic fermentation, sampling was performed at sugar contents of 100%, 80%, 50%, 25%, and 0%. The samples were centrifuged at 2060 x *g* for 5 min for lipid analysis, at 6800 x *g* for 10 min for vitamin analysis, and at 9000 x *g* for 5 min for assimilable nitrogen analysis. The supernatants were collected and stored at -20 °C until analysis.

### Lipidomic profiling

#### Lipid extraction

The lipid analyses were performed at a dedicated lipid analysis platform, DiviOmics, US 58 BioSanD, Université Bourgogne Europe, Dijon, France, specialized in the characterization of lipid compounds by LC-MS and GC-MS. Lipids were extracted using the method of Bligh and Dyer, 1959^74^, adapted to 10 mL glass tubes (Interchim, Montluçon, France), with technical triplicates performed for each extraction. 6 mL of each defrosted sample supernatant was extracted. Each grape juice sample was split into 1.5 mL aliquots and transferred into 10 ml glass tubes. The first set of aliquots was used to measure Total Fatty Acids (TFA), the second for Free Fatty Acids (FFA), the third for Sterols (ST), and the fourth for phospholipids, sterol esters, glycerolipids, and sphingolipids. An internal standard mix 1 (ISTD1, 25 µL) containing a total amount of: C12:0 d_3_, 1.020 µg; C14:0 d_3_, 1.086 µg; C16:0 d_3_, 4.971 µg; C18:0 d_3_, 2.981 µg; C18:2 ω6 d_8_, 0.020 µg; C22:0 d_3_, 0.250 µg; C24:0 d_4_, 0.251 µg; C26:0 d_4_, 0.494 µg) (Cayman, Ann Arbor, USA) was added to the first & second sets of aliquots. Epicoprostanol (5 µg) (Sigma-Aldrich, Saint-Louis, USA), used as sterol ISTD, was added to the third set of aliquots. An internal standard mix 2 (ISTD2, 10 µL) containing a total amount of: (17:0)^2^ d_5_ DG, 200 ng; (17:0)^3^ TG, 250 ng; 14:0 LPE, 150 ng; (17:0)^2^ PE, 500 ng; 14:0 LPC, 75 ng; (19:0)^2^ PC, 500 ng; (21:0)^2^ PC, 500 ng; d18:1/12:0 SM, 500 ng; d18:1/12:0 Cer, 500 ng; 17:0 CE, 200.4 ng) (Avanti Research, Alabaster, USA) was added to the fourth set of aliquots. Extraction was initiated with 5.7 mL of chloroform/methanol (1/2 (v/v)). The solution was vortexed for 5 min. Then 1.9 mL of chloroform and 1.9 mL distilled water (dH2O) were added consecutively with 5 min of shaking between each step. The mixture was centrifuged at 943 x *g* for 5 min. The lower (organic) phase was collected and resuspended in an upper (aqueous) phase prepared by replacing the sample with dH2O. The tubes were vortexed and centrifuged as described previously; the upper phase was removed, and the lower phase was dried under a nitrogen stream at 70°C. Aliquots used to measure SPs, SEs, PLs, and GLs were resuspended with 100 µL of chloroform/methanol/water (60/30/4.5 v/v/v) and directly transferred into vials (Chromoptic, Courtaboeuf, France) with inserts (Sodipro, Echirolles, France) for LC-MS/MS analysis. For TFA and ST quantification, the dried tubes were resuspended and hydrolysed with 1.2 mL of absolute ethanol and 60 µL of 10 M potassium hydroxide, and incubated for 45 min at 56 °C. Then 1 mL of 1.2 M HCl and 3 mL of hexane were added to extract TFAs^75^. STs were extracted with 1 mL of dH_2_O and 5 mL of hexane^76^. For FFA quantification, 1.2 mL of Dole’s reagent (Isopropanol/Hexane/2 M phosphoric acid (40/10/1 v/v) was added to the dried tubes, followed by 1 mL of dH2O, and 1 mL of hexane^77^. The samples were centrifuged for 5 min at 943 x *g*. The hexane phase was recovered and dried under a nitrogen stream at 70 °C.

#### Derivatization for fatty acids and sterols

Fatty Acids (FA) were derivatized with 50 µL of Pentafluorobenzyl bromide /acetonitrile 1:9 v:v (Sigma-Aldrich, Saint-Louis, USA) and 50 µL of N,N-Diisopropylethylamine/acetonitrile 1:9 v:v /(Sigma-Aldrich, Saint-Louis, USA) for 30 min at 37 °C as reported before by Mouillot and colleagues^78^. Derivatized FAs were then extracted with 1 mL of dH2O and 2 mL of hexane. The tubes were centrifuged as before, and the hexane phase was collected and dried under nitrogen at 70 °C. Unlike FAs, STs were derivatized with 100 µL of N, O-Bis(trimethylsilyl)trifluoroacetamide (BSTFA)/Chlorotrimethylsilane (TMCS) (4:1 v:v) (Sigma-Aldrich, Saint-Louis, USA) for 1 hour at 80°C according Scott^79^. Reagents were evaporated under nitrogen and 100 µL of hexane for FA or 100 µL of heptane for ST was used to solubilize the dried lipid extracts prior to GC-MS analysis.

### Targeted analysis

#### Fatty acids

The method and instruments used for total and free fatty acids analysis were adapted from Dumont and colleagues and Mouillot and colleagues^75,78^. The mass spectrometer was operated in Single Ion Monitoring (SIM) mode and masses ranging from 199.2 to 399.40 m/z were acquired (Supplementary data table S1). The method used for sterols analysis was adapted from Thomas and colleagues^76^. GC-MS analyses were performed using a GC Trace 1300 system coupled to a single Quadrupole MS ISQ LT from Thermofisher (Waltham, USA) with an Electronic Impact source. Samples (1 µL) were injected in split mode (ratio of 10). The mass spectrometer was operating in SIM mode according to the mass list described in the Supplementary data table S2.

#### Phospholipids, phytosterol esters, phytoceramides, and glycerides

LC-MS analyses were performed with a 1200 6460-QqQ LC-MS/MS system equipped with Jet Stream Technology (AJS), ESI source (Agilent, Santa Clara, USA). All extracts were analysed by Multiple Reaction Monitoring (MRM). Separation of (lyso)phosphatidylcholines ((L)PC), (lyso)phosphatidylethanolamines ((L)PE), (lyso)phosphatidylinositol (L(PI)), diglycerides (DG) and triglycerides (TG) was performed on an Agilent Zorbax Eclipse Plus C18 2.1 × 100 mm - 1,8 µm column (Santa Clara, USA). On the other hand, ceramides (Cer) and Sterol Esters (SE) were separated on an Agilent Zorbax Eclipse Plus C8 2.1 ×100 mm 1.8 µm column (Santa Clara, USA).

The method for (Lyso)phophatidylcholine and (Lyso)phosphatidylethanolamine analysis used was adapted from Vial and colleagues^80^. For (L)PCs and (L)PEs, 1 and 2 µL of each extract were injected. Positive MRM parameters and source parameters are described in supplementary data, Tables S3 and S4.

As (L)PC and (L)PE, the method used for (Lyso)phosphatidylinositol analysis was adapted from Vial and colleagues^80^. The injection volume was 3 µL, then were separated at 0.4 ml/min with the same mobile phase as phosphatidylcholine and phosphatidylethanolamine according to the following gradient: 20% of B for 2 min, 40% of B for 0.1 min, 65% of B for 11.9 min, 75% of B for 0.1 min, 99% of B for 3.9 min and 20% of B for 6 min. Negative MRM parameters and source parameters are described in supplementary data, table S5.

The method used for phytosterol esters analysis was adapted from Ménégaut and colleagues^81^. Each extract (4 µL) was injected, and lipids were eluted according to the following solvent gradient: 25% of B for 1 min, 70% of B for 13 minutes, 100% of B for 2 min, 25% of B for 0.1 min. Positive MRM parameters and source parameters are described in supplementary data, table S6.

The method used for phytosceramides analysis was adapted from Denimal and colleagues^82^ [65]. Each extract (2 µL) was injected, and lipids were eluted according to the following solvent gradient: 85% of B for 1 min, then 100% for 11 min, then 85% for 0.1 min. The column was kept at 50 °C. Positive MRM parameters and source parameters are described in supplementary data, table S7.

The method used for glycerides analysis was adapted from Cajka and Fiehn^83^. Each extract (4 µL) was injected, and lipids were eluted with a flow rate of 0.400 mL/min according to the following solvent gradient: 30% of B for 1 min, 60% of B for 4 min, 72% of B for 10 min; 99% of B for 12 min, 30% of B for 5 min. Positive MRM parameters and source parameters are described in Supplementary data, Table S8.

### Quantification of lipid adsorption by inactivated yeast

To determine of the amount of lipids that can be adsorbed by yeast, the Chardonnay must be inoculated with an active dried yeast (Oenoferm® Finesse) at a density of 6 × 107 cells/mL, a value chosen to reflect the maximum cell population density reached during alcoholic fermentation. The yeast was rehydrated as described above and inactivated by heat treatment for 15 minutes at 90 °C. The incubation time was 72 hours at 20 °C. Samples were taken at T 0 h and T 72 h for determinations of FFA, TFA, and sterols. The samples were centrifuged at 2060 x *g* for 5 min, frozen and stored at −20 °C until lipid analysis.

### Determination of specific lipid substrate consumption by the yeast

Specific consumption by the yeast was determined for each type of lipid in each set of conditions (strain x must), by calculating the ratio in Equation 1 below:

$${Specific}\,{consumption}=\frac{{\Delta C}_{{lipid}}}{\Delta {population}}* 1\times {10}^{8}\mathrm{viable\; cells}$$

where ${\Delta C}_{{lipid}}$ is the change in lipid concentration between the beginning and end of fermentation (expressed in mg/L) and $\Delta {population}$ is the change in size of the viable yeast population between the beginning and end of t fermentation (expressed in cells/mL). and the resulting ratio represents the concentration of lipid consumed per cell. For readability, results are expressed in mg/L per 1 ×10^8^ viable cells.

### Statistics

Statistical processing and graphical visualizations (volcano plot, bar plot and scatter plot) were performed with R software (Version 4.2.2). Graphical visualizations were performed with *Ggplot2* package (version 3.5.1)^84^. The non-parametric Kruskal-Wallis test (alpha risk = 0.05) was performed with the *Agricolae* package (version 1.3-7)^85^. Principal component analysis was performed with the *FactomineR* package (version 2.11)^86^. The relationship between initial lipid concentration and total specific consumption was assessed by simple linear regression analysis (lm method). Finally, kinetic visualizations and Student’s *t* tests were performed with Excel for Microsoft 365 (version 2209).

## Supplementary information

Supplementary information.

## Data Availability

The authors declare that the data supporting the findings of this study are available within the paper and its Supplementary Information files. Should any raw data files be needed in another format, they are available from the corresponding author upon reasonable request.
