# Supplementary material for: Early and diverse lipid consumption by Saccharomyces cerevisiae: an extensive targeted lipidomics approach and new perspectives for managing wine fermentation
Source: NPJ Sci Food. 2026 Feb 28;10:123. doi: 10.1038/s41538-026-00774-y (PMC13065770; doi:10.1038/s41538-026-00774-y)
Supplement: Supplementary file 1 — Supplementary information. [file 41538_2026_774_MOESM1_ESM.pdf]

Early and Diverse Lipid Consumption by Yeasts: A comprehensive lipidomics approach and New Perspectives for wine fermentation management

Louise Ramousse[1], Jean-Paul Pais de Barros[2,3], Chloé Roullier-Gall[1], Hervé Alexandre[1]

[1] Université Bourgogne Europe, Institut Agro, INRAE, UMR PAM, F-21000 Dijon, France.
[2] UMR1231, Inserm/Université Bourgogne Europe, Dijon, France

[3] Plateforme DiviOmics, US 58 BioSanD, Université Bourgogne Europe, Dijon, France

*Corresponding author: Louise RAMOUSSE, email : louise.ramousse@gmail.com

Supplementary Figure S1 | Growth monitoring of different strains (CX, FE, FI) of Saccharomyces cerevisiae in Chardonnay (A) and Gewurztraminer (B) must at 20 °C. 11
Supplementary Figure S2 | Analysis of the changes in the level of each lipid between the start and end of fermentation, focusing on the strain effect....................13
Supplementary Figure S3 | Mean concentrations of sterols (Sitosterol, Stigmasterol, Campesterol) measured before and after an adsorption assay using inactivated yeast....................14
Supplementary Figure S4 | Changes in mean lipid concentration (mg/L) as a function of residual sugar (%) during the alcoholic fermentation of Chardonnay (left column) and Gewurztraminer (right column) musts. 15
Supplementary Figure S5 | Changes in mean lipid concentration (mg/L) as a function of residual sugar (%) during the alcoholic fermentation of Chardonnay (left column) and Gewurztraminer (right column) musts.. 16
Supplementary Figure S6 | Changes in mean lipid concentration (mg/L) as a function of residual sugar (%) during the alcoholic fermentation of Chardonnay (left column) and Gewurztraminer (right column) musts. 17
Supplementary Figure S7 | Changes in mean lipid concentration (mg/L) as a function of residual sugar (%) during the alcoholic fermentation of Chardonnay (left column) and Gewurztraminer (right column) musts.. 18
Supplementary Figure S8 | Changes in mean lipid concentration (mg/L) as a function of residual sugar (%) during the alcoholic fermentation of Chardonnay (left column) and Gewurztraminer (right column) musts. 19
Supplementary Figure S9 | Changes in mean lipid concentration (mg/L) as a function of residual sugar (%) during the alcoholic fermentation of Chardonnay (left column) and Gewurztraminer (right column) musts. 20
Supplementary Figure S10 | Changes in mean lipid concentration (mg/L) as a function of residual sugar (%) during the alcoholic fermentation of Chardonnay (left column) and Gewurztraminer (right column) musts. 21

Supplementary Table S1 | SIM parameters of Total and Free Fatty Acid analysis....................2
Supplementary Table S2 | SIM parameters of Sterols analysis....................3
Supplementary Table S3 | MRM parameters of (lyso)phosphatidylcholines analysis. 3
Supplementary Table S4 | MRM parameters of (lyso)phosphatidylethanolamines analysis. 5
Supplementary Table S5 | MRM parameters of (lyso)phosphatidylinositols analysis. 6
Supplementary Table S6 | MRM parameters of phytosterol esters analysis....................7
Supplementary Table S7 | MRM parameters of phytoceramides analysis. 8
Supplementary Table S8 | MRM parameters of glycerides analysis. 8
Supplementary Table S9 | Chromatographic gradient for vitamin separation 10
Supplementary Table S10 | Fermentation kinetics parameters of Chardonnay and Gewurztraminer musts with different strains....................11
Supplementary Table S11 | Enological parameters of wines. 11
Supplementary Table S12 | P-values (Student's t-test) for lipids showing significant changes in concentration ($p < 0.05$) before and after fermentation across all experimental conditions.. 12

Supplementary Table S1 | SIM parameters of Total and Free Fatty Acid analysis

Name	Type	m/z	RT(min)	Ion Polarity
C12:0	Target	199.2	13.57	Negative
C12:0 d3	ISTD	202.2	13.523	Negative
C13:0	Target	213.3	15.308	Negative
C14:0	Target	227.2	16.989	Negative
C14:0 d3	ISTD	230.2	16.95	Negative
C14:1	Target	225.2	16.993	Negative
C15:0	Target	241.2	18.637	Negative
C15:1	Target	239.2	18.637	Negative
C16:0	Target	255.2	20.218	Negative
C16:0 d3	ISTD	258.2	20.178	Negative
C16:1 n-7	Target	253.2	19.904	Negative
C16:1 n-9	Target	253.2	19.904	Negative
C17:0	Target	269.2	21.756	Negative
C17:1	Target	267.2	21.343	Negative
C18:0	Target	283.2	23.228	Negative
C18:0 d3	ISTD	286.2	23.195	Negative
C18:1 n-7	Target	281.2	22.805	Negative
C18:1 n-9	Target	281.2	22.848	Negative
C18:2 n-6	Target	279.2	22.792	Negative
C18:2 n-6 d4	ISTD	283.2	22.762	Negative
C18:3 n-3	Target	277.2	22.911	Negative
C18:3 n-6	Target	277.2	22.911	Negative
C19:0	Target	297.3	24.667	Negative
C20:0	Target	311.3	24.912	Negative
C20:0 d3	ISTD	314.3	26.018	Negative
C20:1 n-7	Target	309.3	25.725	Negative
C20:1 n-9	Target	309.3	25.539	Negative
C20:2 n-6	Target	307.3	25.69	Negative
C20:3 n-6	Target	305.2	25.804	Negative
C20:3 n-9	Target	305.2	25.804	Negative
C20:4 n-6	Target	303.2	24.953	Negative
C20:4 n-6 d8	ISTD	311.3	26.052	Negative
C20:5 n-3	Target	301.2	24.963	Negative
C21:0	Target	325.3	27.386	Negative
C22:0	Target	339.3	28.698	Negative
C22:0 d3	ISTD	342.3	28.664	Negative
C22:1 n-7	Target	337.3	27.886	Negative
C22:1 n-9	Target	337.3	27.886	Negative
C22:2 n-6	Target	335.3	27.917	Negative
C22:4 n-6	Target	331.3	27.408	Negative
C22:5 n-3	Target	329.3	27.882	Negative
C22:5 n-6	Target	329.3	27.92	Negative
C22:6 n-3	Target	327.2	27.893	Negative

Name	Type	m/z	RT(min)	Ion Polarity
C23:0	Target	353.3	29.957	Negative
C24:0	Target	367.4	31.18	Negative
C24:0 d4	ISTD	371.4	31.139	Negative
C24:1 n-9	Target	365.3	30.922	Negative
C24:4	Target	359.3	29.88	Negative
C24:5	Target	357.3	29.902	Negative
C24:6 n-3	Target	355.3	29.964	Negative
C25:0	Target	381.4	32.384	Negative
C26:0	Target	395.4	33.553	Negative
C26:0 d4	ISTD	399.4	33.508	Negative
C26:1	Target	393.4	33.326	Negative

Supplementary Table S2 | SIM parameters of Sterols analysis

Name	Type	m/z	RT(min)	Ion Polarity
Ergosterol	Target	363.3	15.79	Positive
Epicoprostanol	ISTD	370.3	13.33	Positive
Campesterol	Target	382.3	16.08	Positive
Sitosterol	Target	396.3	17.34	Positive
Stigmasterol	Target	484.3	16.39	Positive
Stigmastanol	Target	488.3	17.5	Positive

Supplementary Table S3 | MRM parameters of (lyso)phosphatidylcholines analysis. Source parameters was, gas temperature: 325°C, gas flow: 10 L/min, nebulizer: 20 psi, sheath gas heater: 300, sheath gas flow :11, capillary: 4000 V for (L)PC and 3000 V for (L)PE and VCharging: 1000

Name	Transition	Precursor Ion	Product Ion	RT	Ion Polarity	Collision Energy
14:0 LPC	468,5 -> 184,0	468.5	184	2.886	Positive	29
16:0 LPC	496,5 -> 184,0	496.5	184	4.634	Positive	29
16:1 LPC	494,5 -> 184,0	494.5	184	4.6	Positive	29
17:0 LPC	510,4 -> 184,0	510.4	184	5.617	Positive	29
18:0 LPC	524,5 -> 184,0	524.5	184	6.441	Positive	29
18:1 LPC	522,5 -> 184,0	522.5	184	5.177	Positive	29
18:2 LPC	520,5 -> 184,0	520.5	184	4.068	Positive	29
18:3 LPC	518,5 -> 184,0	518.5	184	2.86	Positive	29
19:0 LPC	538,3 -> 184,0	538.3	184	7.255	Positive	29
20:0 LPC	552,5 -> 184,0	552.5	184	8.106	Positive	29
20:1 LPC	550,5 -> 184,0	550.5	184	6.848	Positive	29
20:2 LPC	548,5 -> 184,0	548.5	184	5.765	Positive	29
20:3 LPC	546,5 -> 184,0	546.5	184	4.522	Positive	29
20:4 LPC	544,5 -> 184,0	544.5	184	3.763	Positive	29
22:0 LPC	580,6 -> 184,0	580.6	184	4.465	Positive	29
22:1 LPC	578,6 -> 184,0	578.6	184	2.753	Positive	29
22:2 LPC	576,6 -> 184,0	576.6	184	2.898	Positive	29
22:3 LPC	574,6 -> 184,0	574.6	184	3.192	Positive	29
22:4 LPC	572,5 -> 184,0	572.5	184	2.877	Positive	29
22:5 LPC	570,5 -> 184,0	570.5	184	2.678	Positive	29
22:6 LPC	568,5 -> 184,0	568.5	184	2.403	Positive	29
24:0 PC	622,6 -> 184,1	622.6	184.1	9.597	Positive	33

Name	Transition	Precursor Ion	Product Ion	RT	Ion Polarity	Collision Energy
28:0 PC	678,4 -> 184,1	678.4	184.1	11.077	Positive	33
30:0 PC	706,6 -> 184,1	706.6	184.1	11.945	Positive	33
30:1 PC	704,6 -> 184,1	704.6	184.1	11.621	Positive	33
30:2 PC	702,6 -> 184,1	702.6	184.1	11.112	Positive	33
31:0 PC	720,6 -> 184,1	720.6	184.1	12.524	Positive	33
32:0 PC	734,6 -> 184,1	734.6	184.1	12.635	Positive	33
32:1 PC	732,6 -> 184,1	732.6	184.1	12.957	Positive	33
32:2 PC	730,6 -> 184,1	730.6	184.1	11.993	Positive	33
32:3 PC	728,6 -> 184,1	728.6	184.1	11.587	Positive	33
33:1 PC	746,6 -> 184,1	746.6	184.1	12.957	Positive	33
33:2 PC	744,6 -> 184,1	744.6	184.1	12.461	Positive	33
33:3 PC	742,7 -> 184,1	742.7	184.1	12.013	Positive	33
34:0 PC	762,6 -> 184,1	762.6	184.1	13.549	Positive	33
34:1 PC	760,7 -> 184,1	760.7	184.1	12.841	Positive	33
34:2 PC	758,6 -> 184,1	758.6	184.1	12.345	Positive	33
34:3 PC	756,6 -> 184,1	756.6	184.1	11.869	Positive	33
34:4 PC	754,6 -> 184,1	754.6	184.1	11.634	Positive	33
35:1 PC	774,6 -> 184,1	774.6	184.1	15.763	Positive	33
35:2 PC	772,6 -> 184,1	772.6	184.1	14.722	Positive	33
35:3 PC	770,6 -> 184,1	770.6	184.1	12.873	Positive	33
35:4 PC	768,6 -> 184,1	768.6	184.1	11.977	Positive	33
36:0 PC	790,6 -> 184,1	790.6	184.1	14.693	Positive	33
36:1 PC	788,6 -> 184,1	788.6	184.1	14.025	Positive	33
36:2 PC	786,6 -> 184,1	786.6	184.1	13.136	Positive	33
36:3 PC	784,6 -> 184,1	784.6	184.1	12.55	Positive	33
36:4 PC	782,6 -> 184,1	782.6	184.1	12.04	Positive	33
36:5 PC	780,6 -> 184,1	780.6	184.1	12.156	Positive	33
36:6 PC	778,5 -> 184,1	778.5	184.1	11.687	Positive	33
37:4 PC	796,6 -> 184,1	796.6	184.1	12.782	Positive	33
37:5 PC	794,6 -> 184,1	794.6	184.1	12.548	Positive	33
38:0 PC	818,6 -> 184,1	818.6	184.1	14.678	Positive	33
38:1 PC	816,6 -> 184,1	816.6	184.1	14.292	Positive	33
38:2 PC	814,6 -> 184,1	814.6	184.1	14.056	Positive	33
38:3 PC	812,6 -> 184,1	812.6	184.1	13.642	Positive	33
38:4 PC	810,6 -> 184,1	810.6	184.1	13.443	Positive	33
38:5 PC	808,6 -> 184,1	808.6	184.1	12.898	Positive	33
38:6 PC	806,6 -> 184,1	806.6	184.1	12.458	Positive	33
38:7 PC / 37:0 PC	804,6 -> 184,1	804.6	184.1	12.189	Positive	33
39:1 / 40:8 PC	830,6 -> 184,1	830.6	184.1	12.154	Positive	33
40:3 PC	840,6 -> 184,1	840.6	184.1	14.144	Positive	33
40:4 PC	838,6 -> 184,1	838.6	184.1	13.999	Positive	33
40:5 PC	836,6 -> 184,1	836.6	184.1	13.572	Positive	33
40:6 PC	834,6 -> 184,1	834.6	184.1	13.29	Positive	33
40:7 PC	832,6 -> 184,1	832.6	184.1	12.477	Positive	33
42:0 PC	874,7 -> 184,1	874.7	184.1	15.739	Positive	33

Supplementary Table S4 | MRM parameters of (lyso)phosphatidylethanolamines analysis. Source parameters was, gas temperature: 325°C, gas flow: 10 L/min, nebulizer: 20 psi, sheath gas heater: 300, sheath gas flow :11, capillary: 4000 V for (L)PC and 3000 V for (L)PE and VCharging: 1000

Name	Transition	Precursor Ion	Product Ion	RT	Ion Polarity	Collision Energy
28:0 PE	636,5 -> 495,5	636.5	495.5	11.039	Positive	17
29:0 PE	650,5 -> 509,5	650.5	509.5	11.673	Positive	17
30:0 PE	664,5 -> 523,5	664.5	523.5	12.05	Positive	17
30:1 PE	662,5 -> 521,5	662.5	521.5	11.425	Positive	17
31:0 PE	678,5 -> 537,5	678.5	537.5	14.858	Positive	17
32:0 PE	692,5 -> 551,5	692.5	551.5	12.774	Positive	17
32:1 PE	690,5 -> 549,5	690.5	549.5	12.218	Positive	17
32:2 PE	688,5 -> 547,5	688.5	547.5	11.603	Positive	17
32:3 PE	686,5 -> 545,5	686.5	545.5	11.216	Positive	17
33:0 PE	706,5 -> 565,5	706.5	565.5	13.16	Positive	17
34:0 PE	720,5 -> 579,5	720.5	579.5	13.527	Positive	17
34:1 PE	718,5 -> 577,5	718.5	577.5	13.001	Positive	17
34:2 PE	716,5 -> 575,5	716.5	575.5	12.525	Positive	17
34:3 PE	714,5 -> 573,5	714.5	573.5	12.059	Positive	17
34:4 PE	712,5 -> 571,5	712.5	571.5	11.761	Positive	17
34:5 PE	710,5 -> 569,5	710.5	569.5	10.987	Positive	17
34:6 PE	708,5 -> 567,5	708.5	567.5	10.6	Positive	17
35:1 PE / 36:8	732,5 -> 591,5	732.5	591.5	13.456	Positive	17
35:2 PE	730,5 -> 589,5	730.5	589.5	13	Positive	17
35:3 PE	728,5 -> 587,5	728.5	587.5	12.553	Positive	17
35:4 PE	726,5 -> 585,5	726.5	585.5	11.919	Positive	17
35:5 PE	724,5 -> 583,5	724.5	583.5	13.516	Positive	17
35:6 PE	722,5 -> 581,5	722.5	581.5	13.526	Positive	17
36:0 PE	748,6 -> 607,6	748.6	607.6	14.199	Positive	17
36:1 PE	746,6 -> 605,6	746.6	605.6	13.812	Positive	17
36:2 PE	744,5 -> 603,5	744.5	603.5	13.297	Positive	17
36:3 PE	742,5 -> 601,5	742.5	601.5	12.751	Positive	17
36:4 PE	740,5 -> 599,5	740.5	599.5	12.225	Positive	17
36:5 PE	738,5 -> 597,5	738.5	597.5	11.759	Positive	17
36:6 PE	736,5 -> 595,5	736.5	595.5	11.293	Positive	17
36:7 PE / 35:0	734,5 -> 593,5	734.5	593.5	13.873	Positive	17
38:1 PE	774,5 -> 633,5	774.5	633.5	14.476	Positive	17
38:2 PE	772,6 -> 631,6	772.6	631.6	13.98	Positive	17
38:3 PE	770,6 -> 629,6	770.6	629.6	13.712	Positive	17
38:4 PE	768,5 -> 627,5	768.5	627.5	13.425	Positive	17
38:5 PE	766,5 -> 625,5	766.5	625.5	12.681	Positive	17
38:6 PE	764,5 -> 623,5	764.5	623.5	12.244	Positive	17
39:1 PE	788,5 -> 647,5	788.5	647.5	14.783	Positive	17
39:2 PE	786,5 -> 645,5	786.5	645.5	14.307	Positive	17
39:3 PE	784,5 -> 643,5	784.5	643.5	14.049	Positive	17
39:4 PE	782,6 -> 641,6	782.6	641.6	13.742	Positive	17
40:0 PE	804,5 -> 663,5	804.5	663.5	14.227	Positive	17
40:1 PE	802,6 -> 661,6	802.6	661.6	14	Positive	17
40:2 PE	800,6 -> 659,6	800.6	659.6	14.606	Positive	17
40:3 PE	798,6 -> 657,6	798.6	657.6	14.604	Positive	17
40:4 PE	796,6 -> 655,6	796.6	655.6	13.75	Positive	17
40:5 PE	794,6 -> 653,6	794.6	653.6	13.532	Positive	17
40:6 PE	792,5 -> 651,5	792.5	651.5	13.096	Positive	17
40:7 PE	790,5 -> 649,5	790.5	649.5	12.789	Positive	17

Name	Transition	Precursor Ion	Product Ion	RT	Ion Polarity	Collision Energy
41:1 PE	816,6 -> 675,6	816.6	675.6	15.337	Positive	17
41:2 PE	814,6 -> 673,6	814.6	673.6	14.911	Positive	17
41:3 PE	812,6 -> 671,6	812.6	671.6	14.702	Positive	17
41:5 PE	808,6 -> 667,6	808.6	667.6	13.899	Positive	17
42:0 PE	832,7 -> 691,7	832.7	691.7	15.931	Positive	17
42:1 PE	830,7 -> 689,7	830.7	689.7	15.614	Positive	17
42:2 PE	828,6 -> 687,6	828.6	687.6	14.208	Positive	17
42:3 PE	826,6 -> 685,6	826.6	685.6	14.245	Positive	17
42:4 PE	824,6 -> 683,6	824.6	683.6	14.215	Positive	17
42:5 PE	822,6 -> 681,6	822.6	681.6	13.888	Positive	17
42:6 PE	820,6 -> 679,6	820.6	679.6	13.8	Positive	17
44:1 PE	858,7 -> 717,7	858.7	717.7	16.079	Positive	17
44:2 PE	856,7 -> 715,7	856.7	715.7	15.811	Positive	17
44:3 PE	854,7 -> 713,7	854.7	713.7	15.534	Positive	17
44:4 PE	852,6 -> 711,6	852.6	711.6	15.375	Positive	17
44:5 PE	850,6 -> 709,6	850.6	709.6	14.859	Positive	17
44:6 PE	848,6 -> 707,6	848.6	707.6	14.3	Positive	17
44:7 PE / 43:0	846,7 -> 705,7	846.7	705.7	13.8	Positive	17

Supplementary Table S5 | MRM parameters of (lyso)phosphatidylinositols analysis. Source parameters was, gas temperature: 210°C, gas flow: 11 L/min, nebulizer: 20 psi, sheath gas heater: 350, sheath gas flow: 12, capillary: 4000 V and VCharging : 800.

Name	Transition	Precursor Ion	Product Ion	RT	Ion Polarity	Collision Energy
19:0/19:0 PC	862,7 -> 297,3	862.7	297.3	17.321	Negative	30
21:0/21:0 PC	918,8 -> 325,0	918.8	325	17.991	Negative	50
32:1 PI	807,6 -> 241,0	807.6	241	9.593	Negative	50
34:0 PI	837,6 -> 241,0	837.6	241	13.435	Negative	50
34:1 PI	835,6 -> 241,0	835.6	241	11.963	Negative	50
34:2 PI	833,6 -> 241,0	833.6	241	10.674	Negative	50
34:3 PI	831,6 -> 241,0	831.6	241	9.596	Negative	50
36:0 PI	865,6 -> 241,0	865.6	241	15.068	Negative	50
36:1 PI	863,6 -> 241,0	863.6	241	13.697	Negative	50
36:2 PI	861,6 -> 241,0	861.6	241	12.454	Negative	50
36:3 PI	859,6 -> 241,0	859.6	241	11.306	Negative	50
36:4 PI	857,6 -> 241,0	857.6	241	9.728	Negative	50
36:5 PI	855,6 -> 241,0	855.6	241	8.683	Negative	50
37:4 PI	871,6 -> 241,0	871.6	241	12	Negative	50
38:2 PI	889,6 -> 241,0	889.6	241	14.179	Negative	50
38:3 PI	887,6 -> 241,0	887.6	241	13.075	Negative	50
38:4 PI	885,6 -> 241,0	885.6	241	12.265	Negative	50
38:5 PI	883,6 -> 241,0	883.6	241	11.164	Negative	50
38:6 PI	881,6 -> 241,0	881.6	241	10.675	Negative	50
38:7 PI	879,6 -> 241,0	879.6	241	9.919	Negative	50
40:3 PI	915,6 -> 241,0	915.6	241	15.85	Negative	50
40:4 PI	913,6 -> 241,0	913.6	241	14.794	Negative	50
40:5 PI	911,6 -> 241,0	911.6	241	13.551	Negative	50
40:6 PI	909,6 -> 241,0	909.6	241	11.979	Negative	50
40:7 PI	907,6 -> 241,0	907.6	241	10.719	Negative	50

Supplementary Table S6 | MRM parameters of phytosterol esters analysis. Source parameters was, gas temperature: 150°C, gas flow: 10 L/min, nebulizer: 15 psi, sheath gas heater: 150, sheath gas flow: 11, capillary: 4500 V and VCharging: 1200.

Name	Transition	Precursor Ion	Product Ion	RT	Ion Polarity	Collision Energy
C20:4 CE	690,5 -> 369,3	690.5	369.3	9.915	Positive	17
C28:1-14:0 CASE	628,6 -> 383,4	628.6	383.4	8	Positive	13
C28:1-16:0 CASE	656,6 -> 383,4	656.6	383.4	10.955	Positive	13
C28:1-16:1 CASE	654,6 -> 383,4	654.6	383.4	10.643	Positive	13
C28:1-17:0 CASE	642,6 -> 383,4	642.6	383.4	11	Positive	13
C28:1-17:1 CASE	640,6 -> 383,4	640.6	383.4	11.5	Positive	13
C28:1-18:0 CASE	684,7 -> 383,4	684.7	383.4	11.865	Positive	13
C28:1-18:1 CASE	682,6 -> 383,4	682.6	383.4	11.261	Positive	13
C28:1-18:2 CASE	680,6 -> 383,4	680.6	383.4	10.635	Positive	13
C28:1-18:3 CASE	678,6 -> 383,4	678.6	383.4	9.964	Positive	13
C29:1-14:0 SISE	642,6 -> 397,4	642.6	397.4	10.442	Positive	13
C29:1-16:0 SISE	670,6 -> 397,4	670.6	397.4	11.399	Positive	13
C29:1-16:1 SISE	668,6 -> 397,4	668.6	397.4	11.041	Positive	13
C29:1-17:0 SISE	684,7 -> 397,4	684.7	397.4	11.685	Positive	13
C29:1-17:1 SISE	682,6 -> 397,4	682.6	397.4	11.037	Positive	13
C29:1-18:0 SISE	698,7 -> 397,4	698.7	397.4	12.263	Positive	13
C29:1-18:1 SISE	696,7 -> 397,4	696.7	397.4	11.681	Positive	13
C29:1-18:2 SISE	694,6 -> 397,4	694.6	397.4	11.032	Positive	13
C29:1-18:3 SISE	692,6 -> 397,4	692.6	397.4	10.406	Positive	13
C29:1-20:1 SISE	724,7 -> 397,4	724.7	397.4	12.551	Positive	13
C29:1-20:3 SISE	720,7 -> 397,4	720.7	397.4	11.32	Positive	13
C29:1-21:0 SISE	740,7 -> 397,4	740.7	397.4	13	Positive	13
C29:1-22:0 SISE	754,7 -> 397,4	754.7	397.4	13.827	Positive	13
C29:2 -14:0 STSE	640,6 -> 395,4	640.6	395.4	9.793	Positive	13
C29:2 -16:0 STSE	668,6 -> 395,4	668.6	395.4	10.728	Positive	13
C29:2 -16:1 STSE	666,6 -> 395,4	666.6	395.4	10.349	Positive	13
C29:2 -17:0 STSE	682,6 -> 395,4	682.6	395.4	11.014	Positive	13
C29:2 -17:1 STSE	680,6 -> 395,4	680.6	395.4	10.635	Positive	13
C29:2 -18:0 STSE	696,7 -> 395,4	696.7	395.4	11.681	Positive	13
C29:2 -18:1 STSE	694,6 -> 395,4	694.6	395.4	11.01	Positive	13
C29:2 -18:2 STSE	692,6 -> 395,4	692.6	395.4	10.361	Positive	13
C29:2 -18:3 STSE	690,6 -> 395,4	690.6	395.4	9.735	Positive	13
C29:2 -19:0 STSE	710,7 -> 395,4	710.7	395.4	11	Positive	13
C29:2 -20:1 STSE	722,7 -> 395,4	722.7	395.4	9.348	Positive	13
C29:2 -20:3 STSE	718,6 -> 395,4	718.6	395.4	7.557	Positive	13

Supplementary Table S7 | MRM parameters of phytoceramides analysis. Source parameters was, gas temperature: 325°C, gas flow: 10 L/min, nebulizer: 20 psi, sheath gas heater: 350, sheath gas flow :11, capillary : 3500 V and VCharging : 600.

Name	Transition	Precursor Ion	Product Ion	RT	Ion Polarity	Collision Energy
18:0;O3/16:0(2OH)	572,5 -> 264,3	572.5	264.3	7.334	Positive	25
18:0;O3/18:0(2OH)	600,6 -> 318,3	600.6	318.3	7.662	Positive	25
18:0;O3/18:3(2OH)	594,5 -> 264,3	594.5	264.3	7.866	Positive	25
18:1;O3/22:0(2OH)	654,6 -> 262,3	654.6	262.3	9.104	Positive	25
18:1;O3/23:1(2OH)	668,6 -> 280,3	668.6	280.3	9.393	Positive	25
18:1;O3/24:0(2OH)	682,6 -> 262,3	682.6	262.3	9.663	Positive	25
18:1;O3/25:0(2OH)	696,6 -> 262,3	696.6	262.3	9.934	Positive	25
18:1;O3/26:0(2OH)	692,7 -> 262,3	692.7	262.3	10.166	Positive	35
d18:0/12:0	484,5 -> 266,3	484.5	266.3	6.522	Positive	33
d18:0/24:0	650,7 -> 266,3	650.7	266.3	10.345	Positive	33
d18:1/24:0	632,6 -> 264,3	632.6	264.3	10.423	Positive	25

Supplementary Table S8 | MRM parameters of glycerides analysis. Source parameters was, gas temperature: 150°C, gas flow: 11 L/min, nebulizer: 15 psi, sheath gas heater: 150, sheath gas flow :11, capillary: 4500 V and VCharging: 1200.

Name	Transition	Precursor Ion	Product Ion	RT	Ion Polarity	Collision Energy
12:0_16:0_18:1 TG	794,7 -> 495,4	794.7	495.4	19.999	Positive	25
14:0_16:0_18:1 TG	822,8 -> 523,5	822.8	523.5	20.799	Positive	25
14:0_16:0_18:2 TG	820,7 -> 575,5	820.7	575.5	20.141	Positive	25
14:0_16:0_18:3 TG	818,7 -> 523,5	818.7	523.5	19.483	Positive	25
14:0_18:2_18:2 TG	844,7 -> 547,5	844.7	547.5	19.527	Positive	25
14:0_18:2_18:3 TG	842,7 -> 545,5	842.7	545.5	18.779	Positive	25
14:0_18:3_18:3 TG	840,7 -> 545,5	840.7	545.5	17.89	Positive	25
15:0_16:0_17:0 TG	824,8 -> 565,5	824.8	565.5	21.284	Positive	25
15:0_16:0_18:2 TG	834,8 -> 537,5	834.8	537.5	20.536	Positive	25
15:0_18:3_18:3 TG	854,7 -> 559,5	854.7	559.5	18.599	Positive	25
15:1_16:0_18:2 TG	832,7 -> 535,5	832.7	535.5	20.043	Positive	25
15:1_16:0_18:3 TG	830,7 -> 573,5	830.7	573.5	19.341	Positive	25
15:1_18:2_18:2 TG	856,8 -> 559,5	856.8	559.5	19.384	Positive	25
16:0_16:0 DG	586,5 -> 313,3	586.5	313.3	11.567	Positive	29
16:0_16:0_18:0 TG	852,8 -> 579,5	852.8	579.5	21.963	Positive	25
16:0_16:0_18:1 TG	850,8 -> 577,5	850.8	577.5	21.425	Positive	25
16:0_16:0_18:2 TG	848,8 -> 575,5	848.8	575.5	20.887	Positive	25
16:0_16:0_18:3 TG	846,8 -> 573,5	846.8	573.5	20.342	Positive	25
16:0_17:0_18:2 TG	862,8 -> 589,5	862.8	589.5	21.223	Positive	25
16:0_18:0_18:1 TG	878,8 -> 577,5	878.8	577.5	22.007	Positive	25
16:0_18:1 DG	612,6 -> 313,3	612.6	313.3	11.786	Positive	29
16:0_18:1_18:1 TG	876,8 -> 577,5	876.8	577.5	21.476	Positive	25
16:0_18:1_18:2 TG	874,8 -> 575,5	874.8	575.5	20.953	Positive	25
16:0_18:1_22:0 TG	934,9 -> 577,5	934.9	577.5	22.961	Positive	25
16:0_18:2 DG	610,5 -> 313,3	610.5	313.3	10.621	Positive	29
16:0_18:2_18:2 TG	872,8 -> 575,5	872.8	575.5	20.377	Positive	25
16:0_18:2_18:3 TG	870,8 -> 573,5	870.8	573.5	19.757	Positive	25
16:0_18:2_21:0 TG	918,8 -> 575,5	918.8	575.5	22.363	Positive	25
16:0_18:2_22:0 TG	932,9 -> 575,5	932.9	575.5	22.594	Positive	25
16:0_18:3 DG	608,5 -> 313,3	608.5	313.3	9.679	Positive	29
16:0_18:3_18:3 TG	868,7 -> 573,5	868.7	573.5	19.054	Positive	25
16:0_18:3_22:0 TG	930,9 -> 573,5	930.9	573.5	22.251	Positive	25

Name	Transition	Precursor Ion	Product Ion	RT	Ion Polarity	Collision Energy
17:0_17:0_17:0 TG	866,8 -> 579,5	866.8	579.5	22.217	Positive	25
17:0_17:0-d5 DG	619,6 -> 332,3	619.6	332.3	13.301	Positive	29
17:0_18:2_18:2 TG	886,8 -> 589,5	886.8	589.5	20.75	Positive	25
17:0_18:2_18:3 TG	884,8 -> 587,5	884.8	587.5	20.095	Positive	25
17:1_17:1_17:1 TG	860,8 -> 575,5	860.8	575.5	20.692	Positive	25
17:1_18:1_18:1 TG	888,8 -> 589,5	888.8	589.5	21.288	Positive	25
17:1_18:2_18:3 TG	882,8 -> 585,5	882.8	585.5	19.382	Positive	25
18:0_18:0 DG	642,6 -> 341,3	642.6	341.3	6.493	Positive	29
18:0_18:0_18:1 TG	906,8 -> 605,6	906.8	605.6	22.513	Positive	25
18:0_18:0_18:2 TG	904,8 -> 603,5	904.8	603.5	22.065	Positive	25
18:0_18:1 DG	640,6 -> 341,3	640.6	341.3	13.439	Positive	29
18:0_18:1_18:2 TG	902,8 -> 601,5	902.8	601.5	21.594	Positive	25
18:0_18:2 DG	638,6 -> 341,3	638.6	341.3	12.177	Positive	29
18:0_18:2_22:0 TG	960,9 -> 603,5	960.9	603.5	23.005	Positive	25
18:0_18:3 DG	636,6 -> 341,3	636.6	341.3	11.106	Positive	29
18:0_18:3_22:0 TG	958,9 -> 601,5	958.9	601.5	22.646	Positive	25
18:1_18:1_18:1 TG	902,8 -> 603,5	902.8	603.5	21.541	Positive	25
18:1_18:1_18:2 TG	900,8 -> 603,5	900.8	603.5	21.071	Positive	25
18:1_18:2 DG	636,6 -> 339,3	636.6	339.3	10.815	Positive	29
18:1_18:2_18:2 TG	898,8 -> 601,5	898.8	601.5	20.45	Positive	25
18:2_18:2 DG	634,5 -> 337,3	634.5	337.3	9.757	Positive	29
18:2_18:2_18:2 TG	896,8 -> 599,5	896.8	599.5	19.8	Positive	25
18:2_18:2_18:3 TG	894,8 -> 597,5	894.8	597.5	19.105	Positive	25
18:2_18:2_20:0 TG	928,8 -> 631,6	928.8	631.6	21.698	Positive	25
18:2_18:2_21:0 TG	942,8 -> 645,6	942.8	645.6	21.974	Positive	25
18:2_18:2_22:0 TG	956,9 -> 659,6	956.9	659.6	22.235	Positive	25
18:2_18:3 DG	632,5 -> 337,3	632.5	337.3	8.899	Positive	29
18:2_18:3_18:3 TG	892,7 -> 597,5	892.7	597.5	18.283	Positive	25
18:2_18:3_20:0 TG	926,8 -> 597,5	926.8	597.5	21.257	Positive	25
18:2_18:3_22:0 TG	954,8 -> 597,5	954.8	597.5	21.861	Positive	25
18:3_18:3 DG	630,5 -> 335,3	630.5	335.3	8.138	Positive	29
18:3_18:3_18:3 TG	890,7 -> 595,5	890.7	595.5	17.296	Positive	25
18:3_18:3_22:0 TG	952,8 -> 595,5	952.8	595.5	21.413	Positive	25

Supplementary Table S9 | Chromatographic gradient for vitamin separation

20min 0,6 mL/min			
**Time (min)**	**Phosphoric Acid (%)**	**Methanol (%)**	**Flow rate (ml/min)**
0	100	0	0,6
5	100	0	0,6
15	90	10	0,6
36	75	25	0,6
47	67	33	0,6
67	75	25	0,6
70	100	0	0,6
80	100	0	0,6

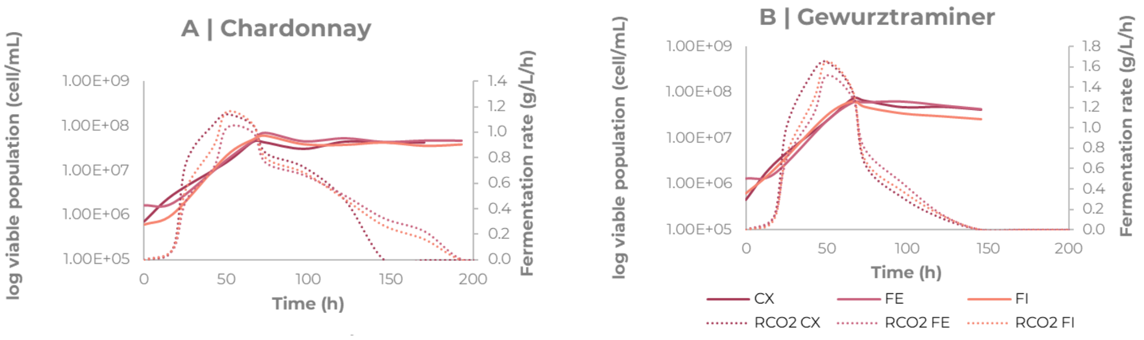

Supplementary Figure S1 | Growth monitoring of different strains (CX, FE, FI) of Saccharomyces cerevisiae in Chardonnay (A) and Gewurztraminer (B) must at 20 °C. Solid lines represent the mean of the evolution of yeast cell concentration (log scale, cells/mL). Dotted lines represent the mean of CO_2 production rate (g/L/h) for the corresponding treatment. The "RCO_2_" prefix denotes the CO_2 production rate for each respective condition.

Supplementary Table S10 | Fermentation kinetics parameters of Chardonnay and Gewurztraminer musts with different strains. The parameters include the maximum specific growth rate (μmax, h^{-1}), the maximum yeast population (cells/mL), the generation time (G time, h), the total fermentation time (fermentation time, h) and the maximum CO_2 production rate (RCO_2max, g/L/h). Data are expressed as mean ± standard deviation (n = 3).

Samples	μmax h^{-1}	Maximum population Cells/ml	G time h	Fermentation time h	RCO_2max g/L/h
CH CX	0.2 ± 0	5.13E+07 ± 1.06E+07	3.5 ± 0.4	170 ± 0	1.1 ± 0
CH FE	0.2 ± 0.1	7.02E+07 ± 9.25E+06	3.5 ± 1.1	193 ± 0	1 ± 0.1
CH FI	0.3 ± 0.1	6.51E+07 ± 1.11E+07	2.8 ± 1.2	193 ± 0	1.2 ± 0
GE CX	0.2 ± 0	7.27E+07 ± 4.59E+06	3.4 ± 0.6	146 ± 0	1.6 ± 0
GE FE	0.2 ± 0.1	6.91E+07 ± 2.59E+06	3 ± 0.9	146 ± 0	1.5 ± 0
GE FI	0.2 ± 0	6.30E+07 ± 5.10E+06	3.6 ± 0.5	146 ± 0	1.6 ± 0

Supplementary Table S11 | Enological parameters of wines. Parameters were measured after alcoholic fermentation using FTIR and sugar by enzymatic analysis (n=3).

Samples	Acohol content %	Glucose /fructose g/L	Total Acidity g/L	pH	Volatile Acidity g/L	Malic Acid g/L	Tartaric Acid g/L	Density g/mL
CH CX	14.38 ± 0.01	1.42 ± 0.1	3.78 ± 0.02	3.45 ± 0.01	0.11 ± 0.01	3.34 ± 0.02	1.3 ± 0.02	0.99 ± 0
CH FE	14 ± 0.01	1.48 ± 0.22	4.35 ± 0.04	3.38 ± 0	0.1 ± 0.01	3.67 ± 0.02	1.05 ± 0.04	0.99 ± 0
CH FI	13.99 ± 0.06	1.56 ± 0.11	4.14 ± 0.05	3.39 ± 0.01	0.09 ± 0.01	3.62 ± 0.03	1.1 ± 0.03	0.99 ± 0
GE CX	12.8 ± 0.02	0.34 ± 0.11	3.83 ± 0.01	3.54 ± 0.01	0.09 ± 0.01	4 ± 0.02	1.36 ± 0.02	0.99 ± 0
GE FE	12.66 ± 0.01	0.29 ± 0.06	4.14 ± 0.01	3.49 ± 0.01	0.26 ± 0.01	4.07 ± 0.02	1.3 ± 0.02	0.99 ± 0
GE FI	12.7 ± 0.02	0.44 ± 0.06	4.05 ± 0.01	3.48 ± 0.01	0.23 ± 0.01	4.12 ± 0.01	1.36 ± 0.03	0.99 ± 0

Supplementary Table S12 | P-values (Student's t-test) for lipids showing significant changes in concentration ($p < 0.05$) before and after fermentation across all experimental conditions. Analyses were conducted on two grape varieties (Chardonnay and Gewurztraminer) and with three yeast strains (CX9, Fermol, and Finesse).

	**Chardonnay**			**Gewurztraminer**		
**Lipid spcies**	**CX9**	**Fermol**	**Finesse**	**CX9**	**Fermol**	**Finesse**
16:0_16:0 DG	0.001	0.000	0.005			
16:0_18:1 DG	1.69E-05	4.56E-05	2.29E-04	1.63E-04	1.64E-04	1.70E-04
16:0_18:2 DG	6.61E-06	0.005	4.56E-06	4.72E-04	2.45E-04	2.61E-05
16:0_18:3 DG	0.004		1.01E-04	4.27E-04	3.47E-04	1.76E-04
18:0_18:0 DG			0.022			
18:0_18:1 DG	1.82E-04	0.002	0.004	0.028	2.03E-04	0.022
18:0_18:2 DG	0.004	0.011	2.87E-04	0.001	0.001	6.67E-05
18:0_18:3 DG	0.001	0.014	2.08E-04	0.001	2.57E-04	0.001
18:1_18:2 DG	0.013	5.92E-07	0.007	3.76E-04	0.001	0.001
18:2_18:2 DG	2.52E-07	0.002	2.18E-06	3.38E-04	3.05E-04	2.16E-04
18:2_18:3 DG	0.001	0.019	1.31E-04	3.28E-04	0.001	0.001
18:3_18:3 DG	0.046	0.009	0.045	2.65E-04	3.85E-04	0.001
C12:0			0.014		0.001	0.041
C13:0	0.037			0.013		0.001
C14:1		0.022			0.001	1.72E-05
C15:0	0.004	0.004	0.004	0.001	0.001	0.001
C15:1	0.005	0.002		0.001		2.89E-04
C16:0	0.037			0.005		0.006
C16:1 n-7	1.54E-04	2.12E-04	0.002	0.001	2.88E-04	0.001
C16:1 n-9	7.58E-05	8.36E-05	0.028	0.001	0.001	0.001
C17:0	6.70E-05	7.39E-05		1.84E-04	0.041	4.21E-04
C17:1	3.37E-04	3.37E-04	0.000	0.010		0.010
C18:0	0.001	0.001	0.001	0.001	0.001	0.001
C18:1 n-7	0.001	0.001	2.68E-04	0.001	0.001	0.001
C18:1 n-9	0.003	0.005		0.001		0.001
C18:3 n-3					0.015	0.006
C18:3 n-6	0.004	0.001				3.00E-05
C19:0	0.001	0.001				
C20:0	0.013	0.013	0.013	2.69E-04	2.69E-04	2.69E-04
C20:1 n-7	0.013	0.013	0.013	2.69E-04	2.69E-04	2.69E-04
C20:1 n-9	0.010	0.018				
C20:4 n-6	0.001	0.001	0.001	0.002	0.002	0.002
C22:5 n-6	0.004	6.53E-07		0.005	0.003	0.001
C22:6 n-3	0.001	1.26E-04	0.002	0.001	4.76E-05	0.001
C24:5	0.002	1.04E-04		0.004	0.019	0.001
C24:6 n-3	0.002	2.69E-05		0.027	0.009	0.001
C26:0	0.002	2.09E-04	0.002	0.003	3.18E-05	4.60E-05
C26:1	0.001	2.44E-04	0.003	0.011	0.006	0.002
C28:1-14:0 CASE	0.015	0.032	0.032	0.015	0.015	0.015
C28:1-16:0 CASE	0.007	0.009			0.040	
C28:1-18:1 CASE	0.013	0.006	0.005	8.54E-05	1.66E-04	5.28E-05
C28:1-18:2 CASE	0.001	1.06E-05	1.19E-05	0.001	4.08E-04	6.21E-05
C28:1-18:3 CASE	0.026	0.016	0.020	2.16E-04	1.55E-04	0.002
C29:1-14:0 SISE				0.009	0.001	0.002
C29:1-16:0 SISE	0.027	0.015	0.021	2.29E-04	4.56E-04	8.12E-06
C29:1-16:1 SISE	0.048	0.042	0.036	0.001	0.001	0.003
C29:1-17:0 SISE	1.51E-04	0.014	2.04E-04	0.001	3.05E-04	0.007
C29:1-17:1 SISE	0.031	0.018	0.022	0.001	1.39E-04	3.16E-04

Lipid spcies	Chardonnay			Gewurztraminer		
	CX9	Fermol	Finesse	CX9	Fermol	Finesse
C29:1-18:0 SISE	0.007	2.29E-04	0.003	0.002	0.001	
C29:1-18:1 SISE	0.004	4.10E-05	1.88E-04	6.22E-05	8.78E-05	0.001
C29:1-18:2 SISE	0.026	0.011	0.017	0.001	3.53E-04	3.94E-04
C29:1-18:3 SISE	0.025	0.013	0.018	2.14E-04	9.21E-05	0.001
C29:1-20:1 SISE	0.049	0.049	0.049	0.002	0.004	0.003
C29:2 -16:0 STSE	0.003	0.003	0.002	0.001	4.43E-04	0.003
C29:2 -16:1 STSE	0.021	0.024	0.004	0.003	0.001	4.21E-04
C29:2 -17:0 STSE	0.018	0.019	0.019			
C29:2 -17:1 STSE	0.007	0.032	0.002	0.004	1.42E-04	0.001
C29:2 -18:0 STSE	0.002	0.018	0.018	1.08E-04	0.001	5.46E-05
C29:2 -18:1 STSE	0.004	1.99E-04	0.001	0.001	1.36E-04	0.001
C29:2 -18:2 STSE	0.013	0.002	0.003	3.69E-04	1.62E-04	0.002
C29:2 -18:3 STSE	2.24E-06	1.77E-04	1.24E-04	2.25E-04	1.64E-04	0.001
C29:2 -19:0 STSE	0.041	0.038	0.039	0.004	0.001	0.005
Campesterol	0.006	0.001	0.018	1.64E-04	0.001	0.001
Ergosterol	1.03E-05	0.001	2.99E-05	0.040	0.002	0.003
Sitosterol	0.001	0.001	0.007	6.45E-05	2.80E-08	1.50E-05
Stigmastanol	1.57E-05	1.08E-04	9.61E-05	0.002	0.002	0.002
Stigmasterol	0.004	0.003	0.003	0.002	0.002	0.002

Lipids not listed showed no detectable signal or no significant variation.

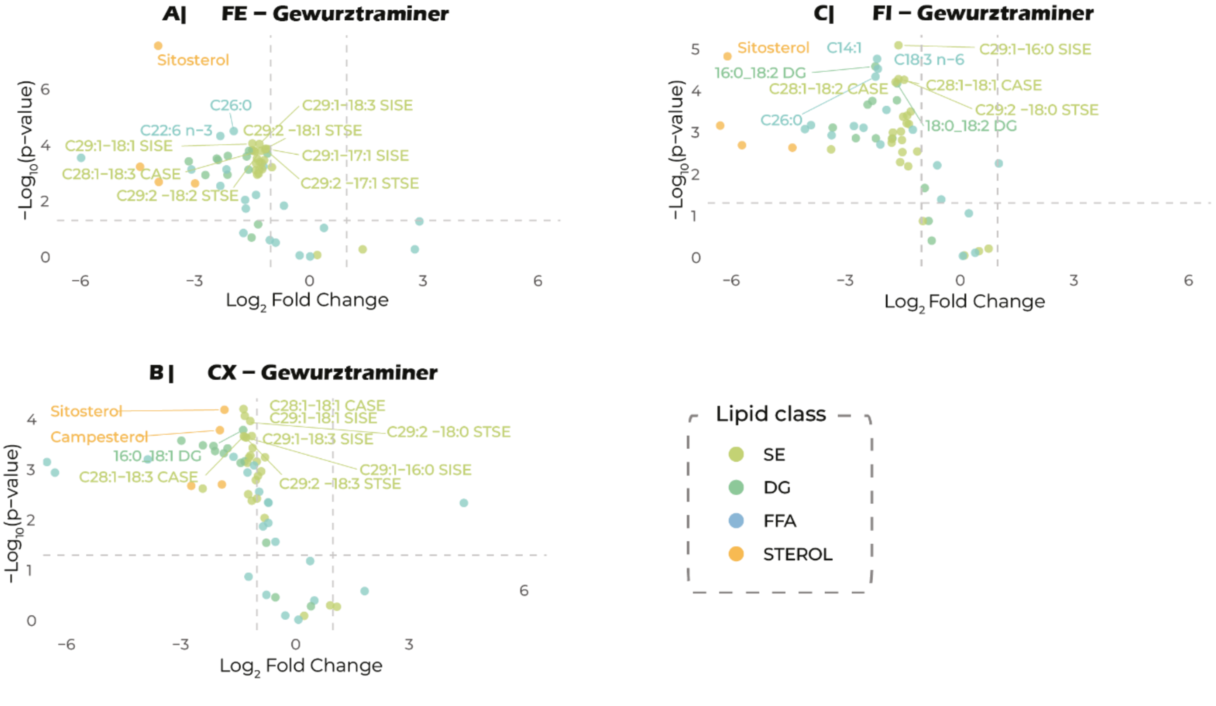

Supplementary Figure S2 | Analysis of the changes in the level of each lipid between the start and end of fermentation, focusing on the strain effect. Volcano plots represent changes in lipid concentration between the start and end of the fermentation for each strain in the Chardonnay must. A: Fermol strain and Chardonnay must, B: CX9 strain and Chardonnay must, and C: Finesse strain and Chardonnay must. The x-axis indicates log_2 fold change, and the y-axis statistical significance ($-log_{10}$ p-value). Dotted lines delimit the thresholds of significance ($p < 0.05$) and biological change ($|log_2FC| > 1$). The annotated lipids are those displaying the greatest change in concentration. Abbreviations: SE: sterol esters, DG: diglycerides, FFA: free fatty acids, CASE: campesterol esters, SISE: sitosterol esters, STSE: stigmastanol esters.

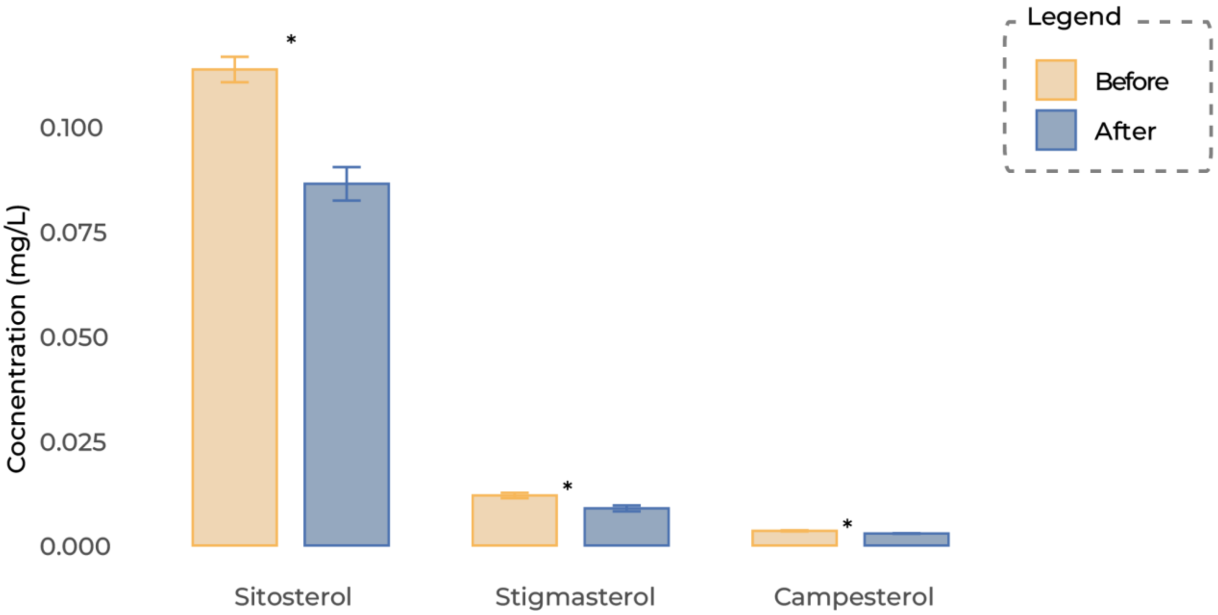

Supplementary Figure S3 | Mean concentrations of sterols (Sitosterol, Stigmasterol, Campesterol) measured before and after an adsorption assay using inactivated yeast. Bars represent the mean values from biological triplicates (n = 3), with error bars indicating standard deviations. Statistical significance between "before" and "after" conditions was assessed using Student's t-test ($p < 0.05$). Asterisks (*) *denote significant differences.*

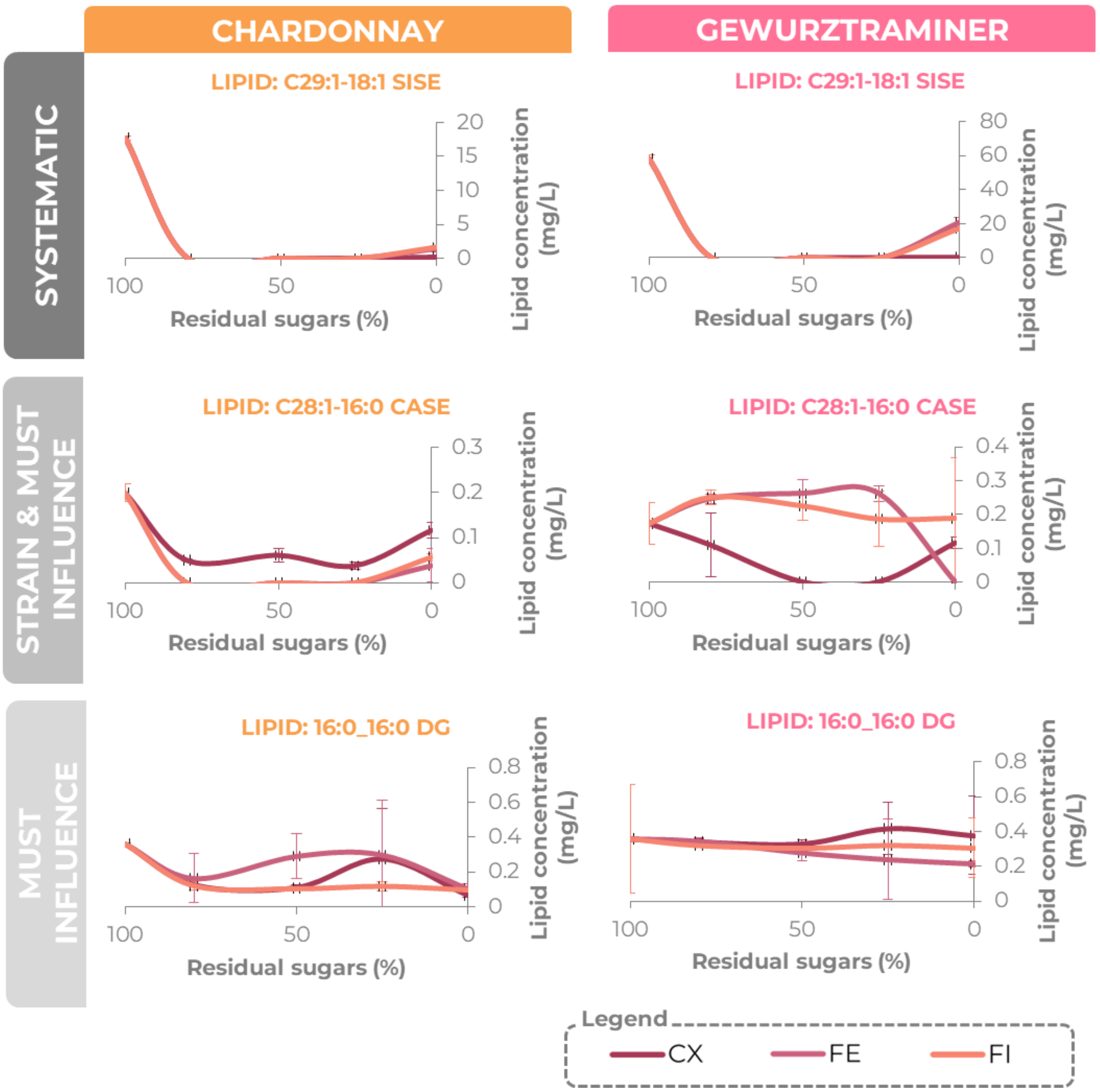

Supplementary Figure S4 | Changes in mean lipid concentration (mg/L) as a function of residual sugar (%) during the alcoholic fermentation of Chardonnay (left column) and Gewurztraminer (right column) musts. This figure includes only combinations in which lipid concentrations changed significantly during the course of fermentation (t-test, α=0.05). Each curve represents a strain: CX (burgundy), FE (pink) and FI (orange). The lipids chosen to illustrate the various influences encountered during the experiment are classified according to the type of influence observed. Systematic: similar profile regardless of strain or medium; strain and matrix influence: variable profiles depending on grape variety and strain; and strain influence: different profiles between strains regardless of grape variety. Error bars represent the standard deviation of the measured values (n=3).

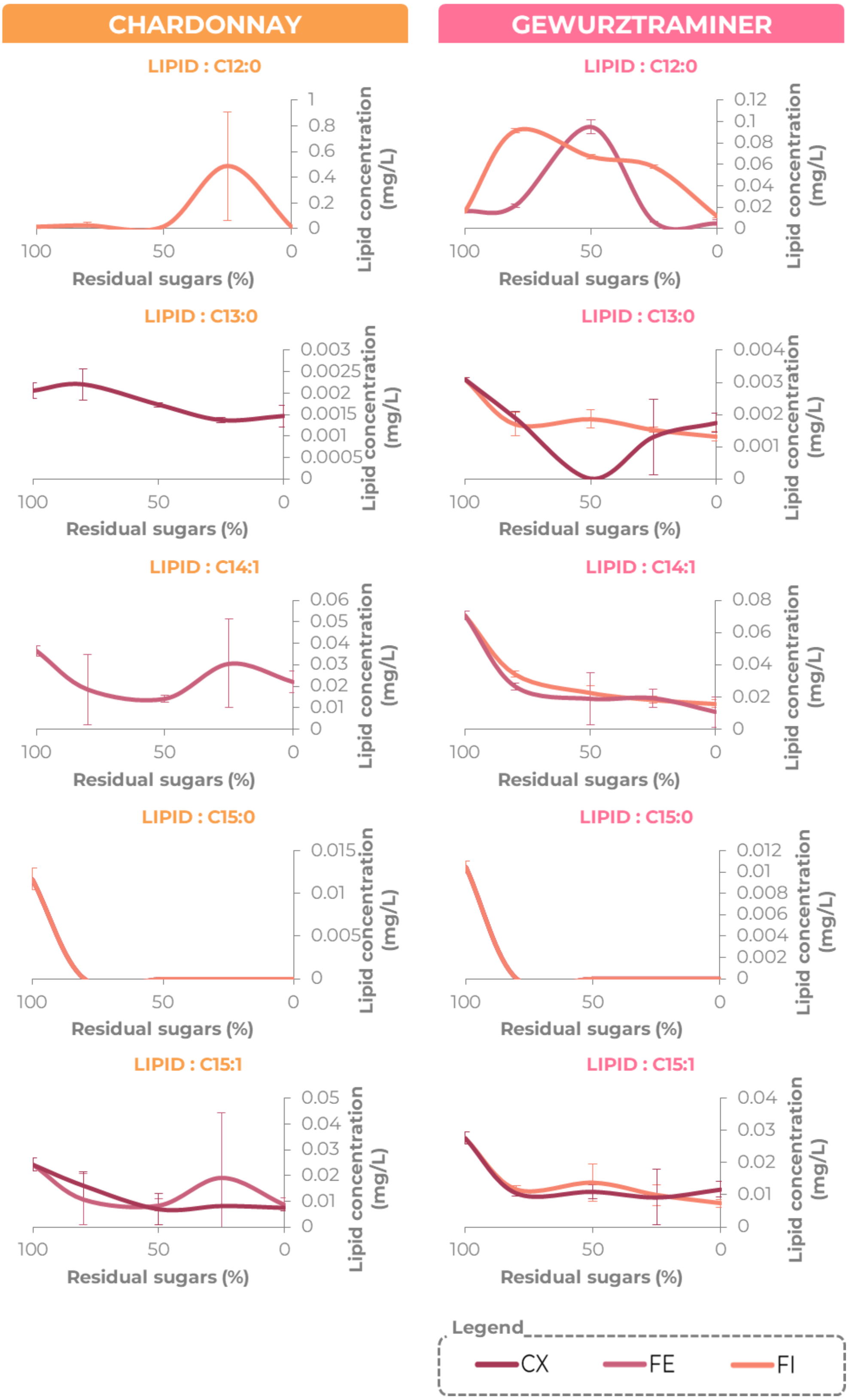

Supplementary Figure S5 | Changes in mean lipid concentration (mg/L) as a function of residual sugar (%) during the alcoholic fermentation of Chardonnay (left column) and Gewurztraminer (right column) musts. This figure includes only combinations in which lipid concentrations changed significantly during the course of fermentation (t-test, α=0.05). Each curve represents a strain: CX (burgundy), FE (pink) and FI (orange). Error bars represent the standard deviation of the measured values (n=3).

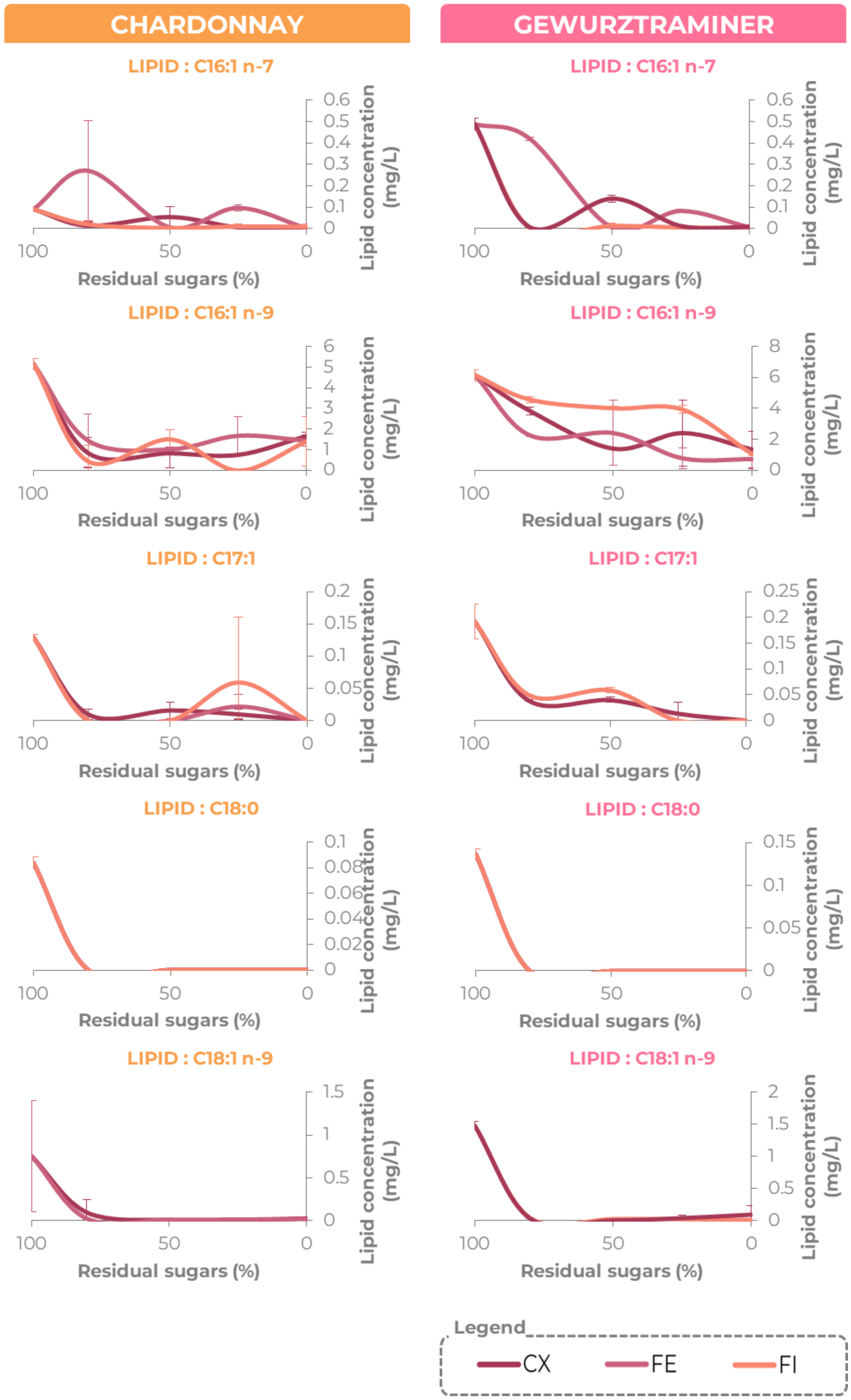

Supplementary Figure S6 | Changes in mean lipid concentration (mg/L) as a function of residual sugar (%) during the alcoholic fermentation of Chardonnay (left column) and Gewurztraminer (right column) musts. This figure includes only combinations in which lipid concentrations changed significantly during the course of fermentation (t-test, α=0.05). Each curve represents a strain: CX (burgundy), FE (pink) and FI (orange). Error bars represent the standard deviation of the measured values (n=3).

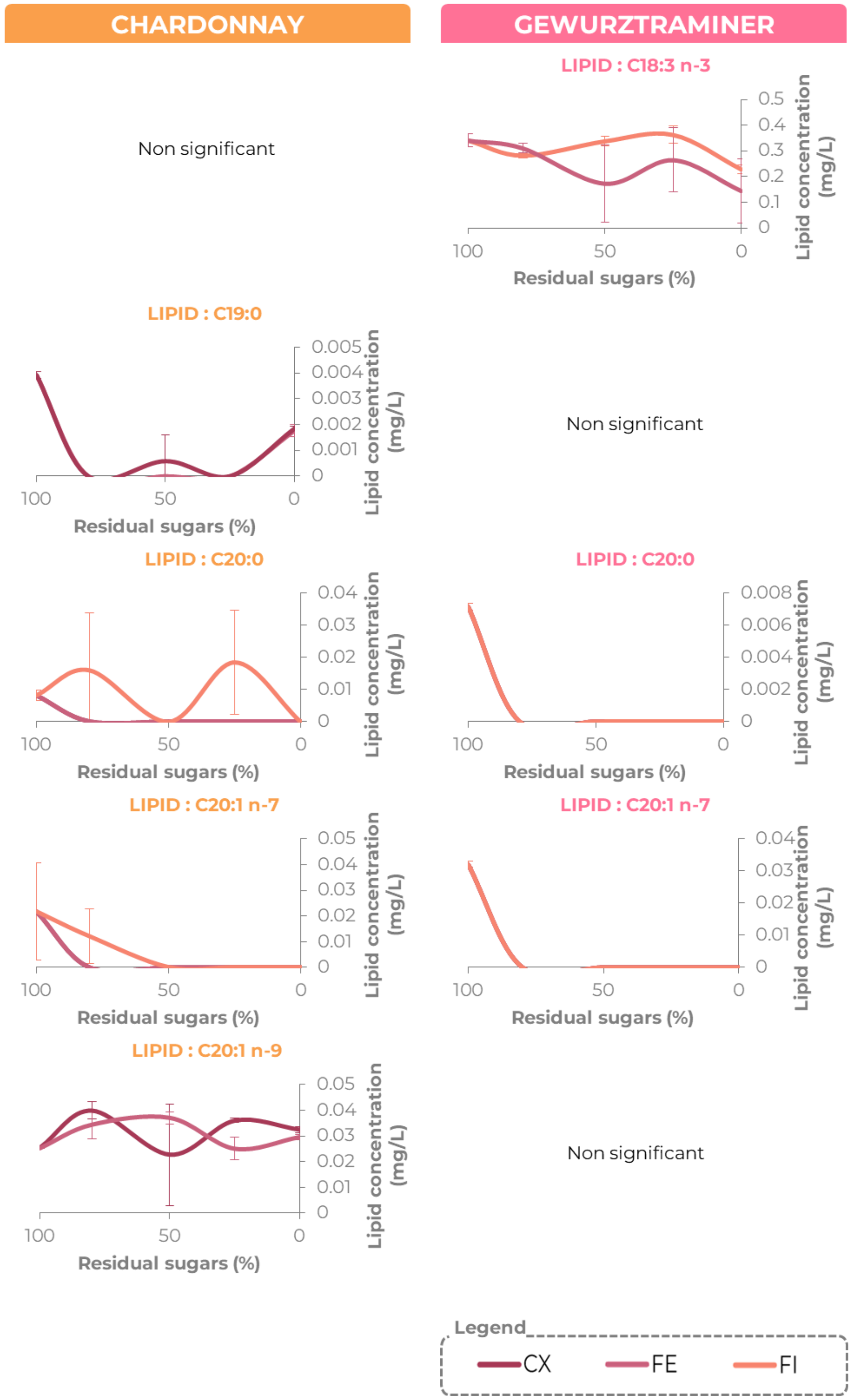

Supplementary Figure S7 | Changes in mean lipid concentration (mg/L) as a function of residual sugar (%) during the alcoholic fermentation of Chardonnay (left column) and Gewurztraminer (right column) musts. This figure includes only combinations in which lipid concentrations changed significantly during the course of fermentation (t-test, α=0.05). Each curve represents a strain: CX (burgundy), FE (pink) and FI (orange). Error bars represent the standard deviation of the measured values (n=3).

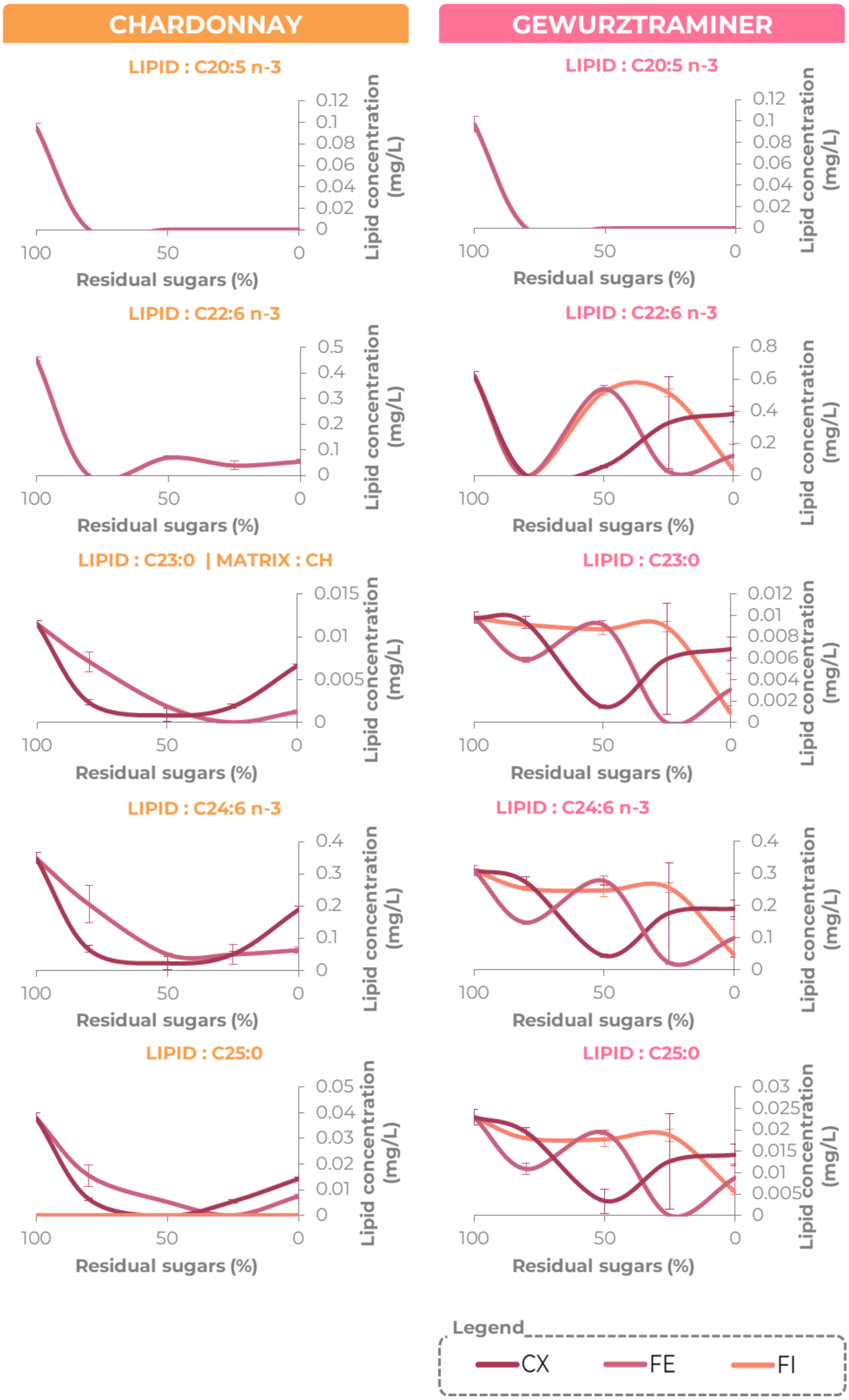

Supplementary Figure S8 | Changes in mean lipid concentration (mg/L) as a function of residual sugar (%) during the alcoholic fermentation of Chardonnay (left column) and Gewurztraminer (right column) musts. This figure includes only combinations in which lipid concentrations changed significantly during the course of fermentation (t-test, α=0.05). Each curve represents a strain: CX (burgundy), FE (pink) and FI (orange). Error bars represent the standard deviation of the measured values (n=3).

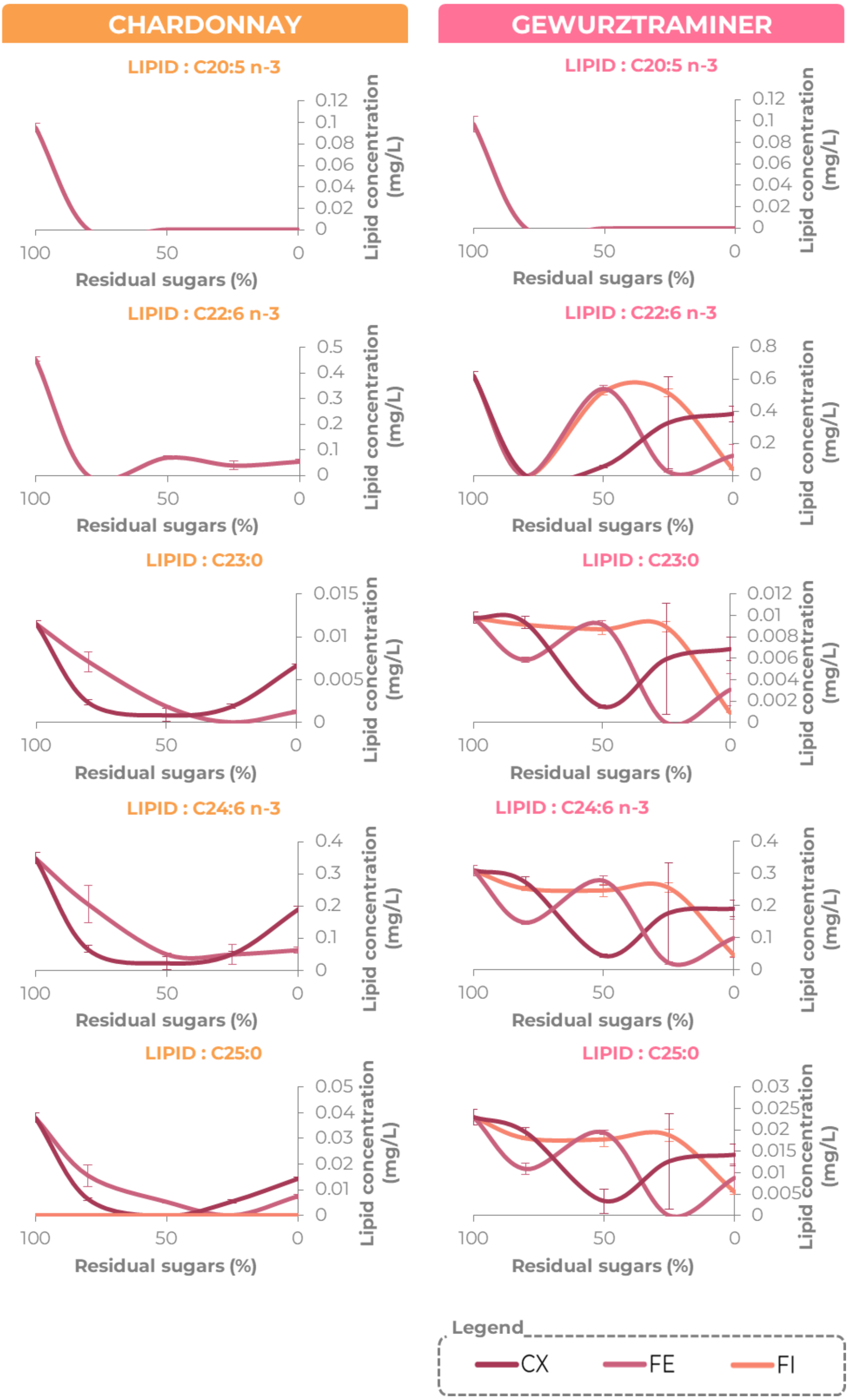

Supplementary Figure S9 | Changes in mean lipid concentration (mg/L) as a function of residual sugar (%) during the alcoholic fermentation of Chardonnay (left column) and Gewurztraminer (right column) musts. This figure includes only combinations in which lipid concentrations changed significantly during the course of fermentation (t-test, α=0.05). Each curve represents a strain: CX (burgundy), FE (pink) and FI (orange). Error bars represent the standard deviation of the measured values (n=3).

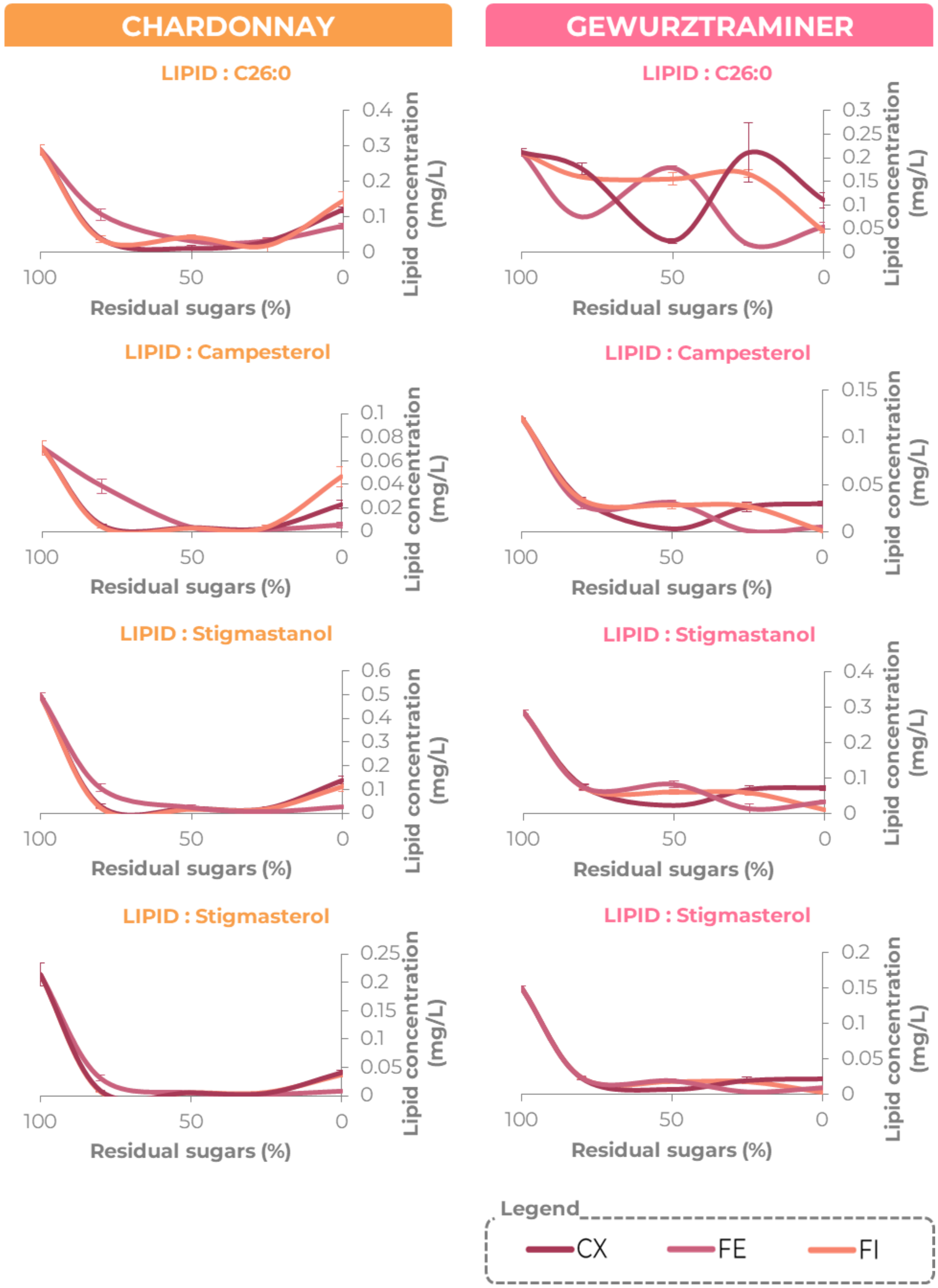

Supplementary Figure S10 | Changes in mean lipid concentration (mg/L) as a function of residual sugar (%) during the alcoholic fermentation of Chardonnay (left column) and Gewurztraminer (right column) musts. This figure includes only combinations in which lipid concentrations changed significantly during the course of fermentation (t-test, α=0.05). Each curve represents a strain: CX (burgundy), FE (pink) and FI (orange). Error bars represent the standard deviation of the measured values (n=3).
